# Supplementary material for: High‐Throughput Screen of NPQ in Sorghum Shows Highly Polygenic Architecture of Photoprotection
Source: Plant Environ Interact. 2026 Jan 11;7(1):e70114. doi: 10.1002/pei3.70114 (PMC12791165; doi:10.1002/pei3.70114)
Supplement: Supplementary file 1 — Figure S1: Plots of maximum daily temperature and total daily precipitation recorded at the Willard Airport weather station (Savoy, IL, USA) during the 2017 and 2019 sorghum panel growing seasons. Figure S2: Plots of daily accumulated solar irradiation recorded at the Illinois State Water Research Center (Champaign, IL, USA) during the 2017 and 2019 sorghum panel growing seasons. Figures S3–S6: Chromosome mapping (Manhattan) plots for single‐nucleotide polymorphisms associated with NPQ and combined NPQ traits in 2017, 2019, and joint genome‐wide association study analyses. Figures S7–S12: Chromosome mapping (Manhattan) plots for genes associated with NPQ and combined NPQ traits in 2017, 2019, and joint transcriptome‐wide association study analyses in third leaf and growing point tissue. Figures S13–S18: Chromosome mapping (Manhattan) plots for genes associated with NPQ and combined NPQ traits in 2017, 2019, and joint Fisher's combined test analyses in third leaf and growing point tissue. Figures S19–S22: QQ plots of GWAS SNPs for 2017, 2019, and joint NPQ traits. Figures S23–S25: Signed Z‐score histograms of univariate genome‐wide association analyses, faceted by trait. Figure S26: Biplot of first two principle components of GWAS SNP set with top Multi‐trait Score accessions highlighted. [file PEI3-7-e70114-s001.pdf]

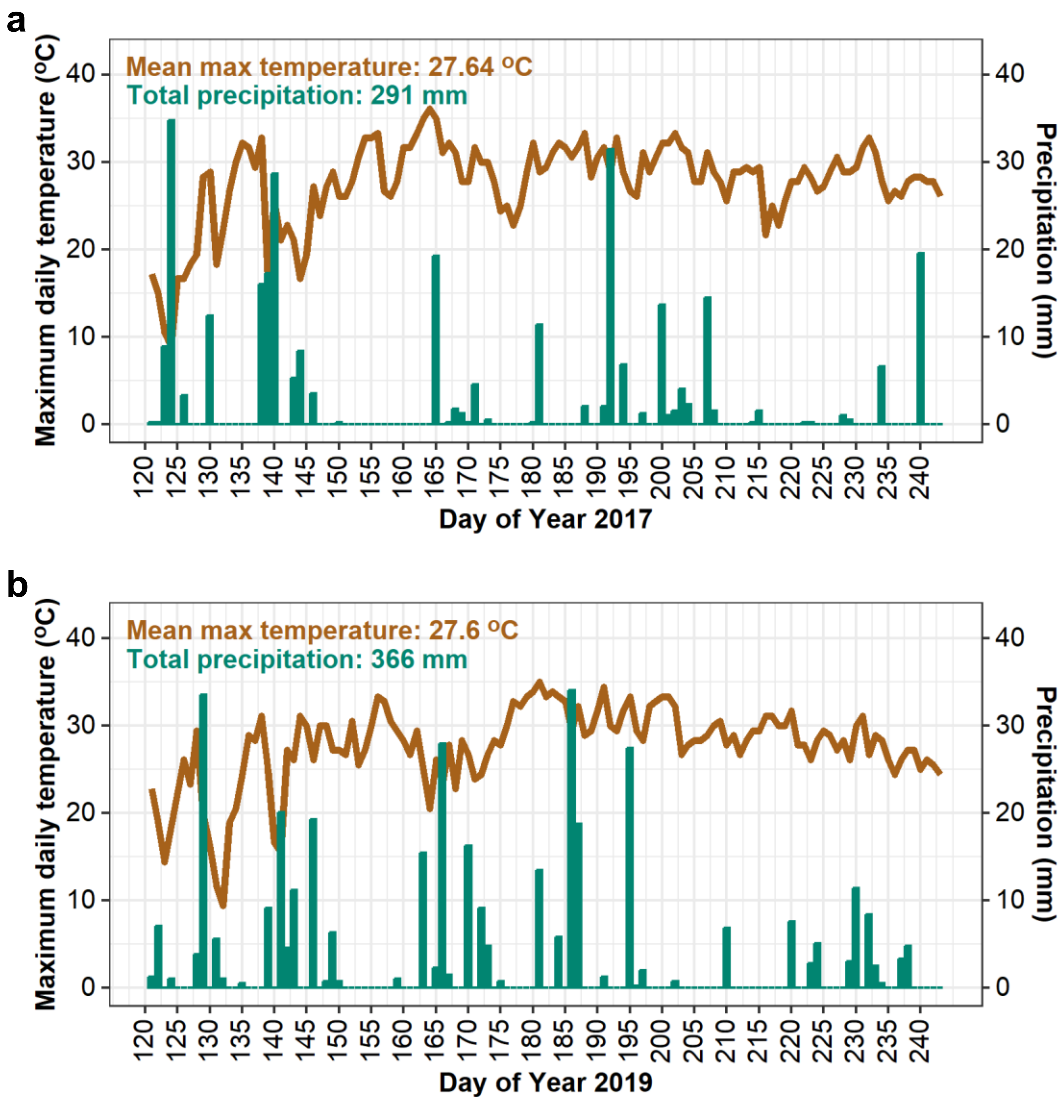

Figure S1: Plots of maximum daily temperature (brown lines) and total daily precipitation (green columns) recorded at the Willard Airport weather station (Savoy, IL, USA) during the 2017 (a) **and 2019** (b) **sorghum panel growing seasons. Seasonal totals calculated from May 05 through August 31. Planting date: 151. Sampling dates: 206-216 (2017); 203-212 (2019). Data retrieved from www.ncdc.noaa.gov (Station ID GHCND:USW00094870; 3.1 km from Maxwell Farm and 6.73 km from Energy Farm).**

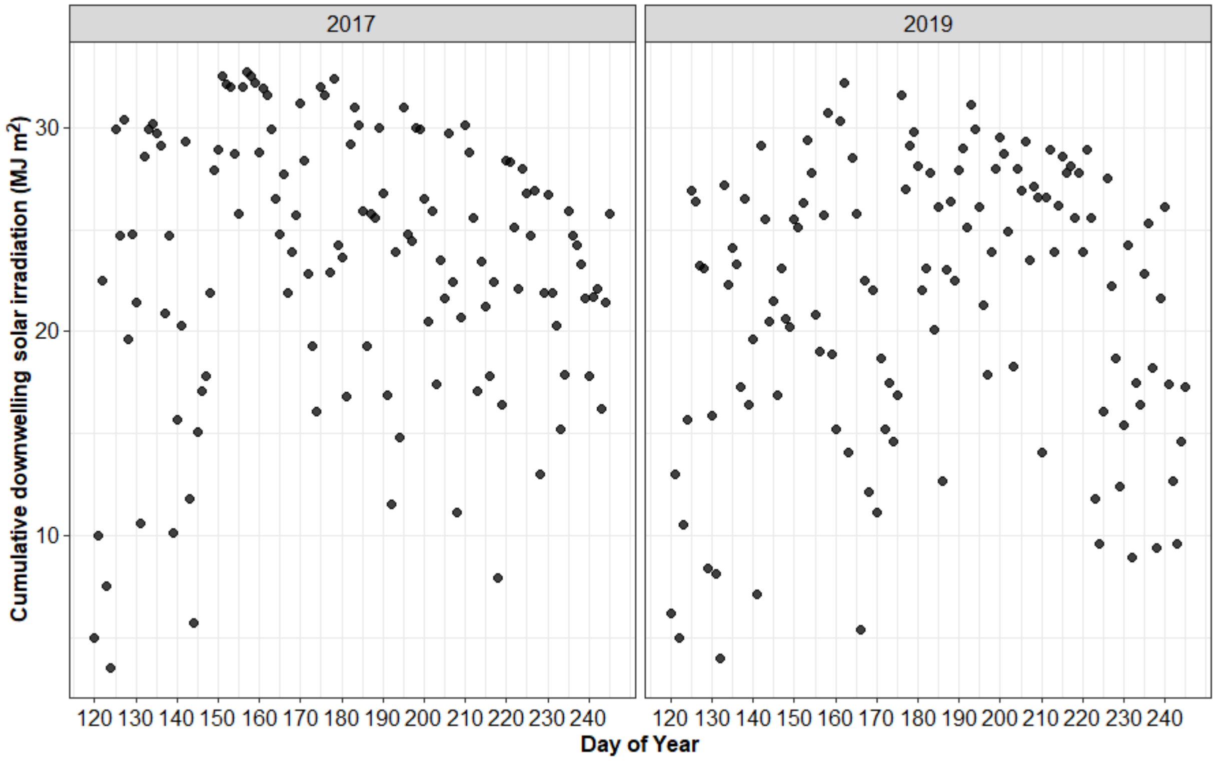

Figure S2: Plots of daily accumulated solar irradiation recorded at the Illinois State Water Research Center (Champaign, IL, USA) during the 2017 and 2019 sorghum panel growing seasons. Planting date: 151. Sampling dates: 206-216 (2017); 203-212 (2019). Cumulative irradiation from day-of-year 151 through 201 was 1,352.4 MJm^2 in 2017 and 1,193.5 MJm^2 in 2019. Data retrieved from Illinois Climate Network (Illinois State Water Survey, 2025).

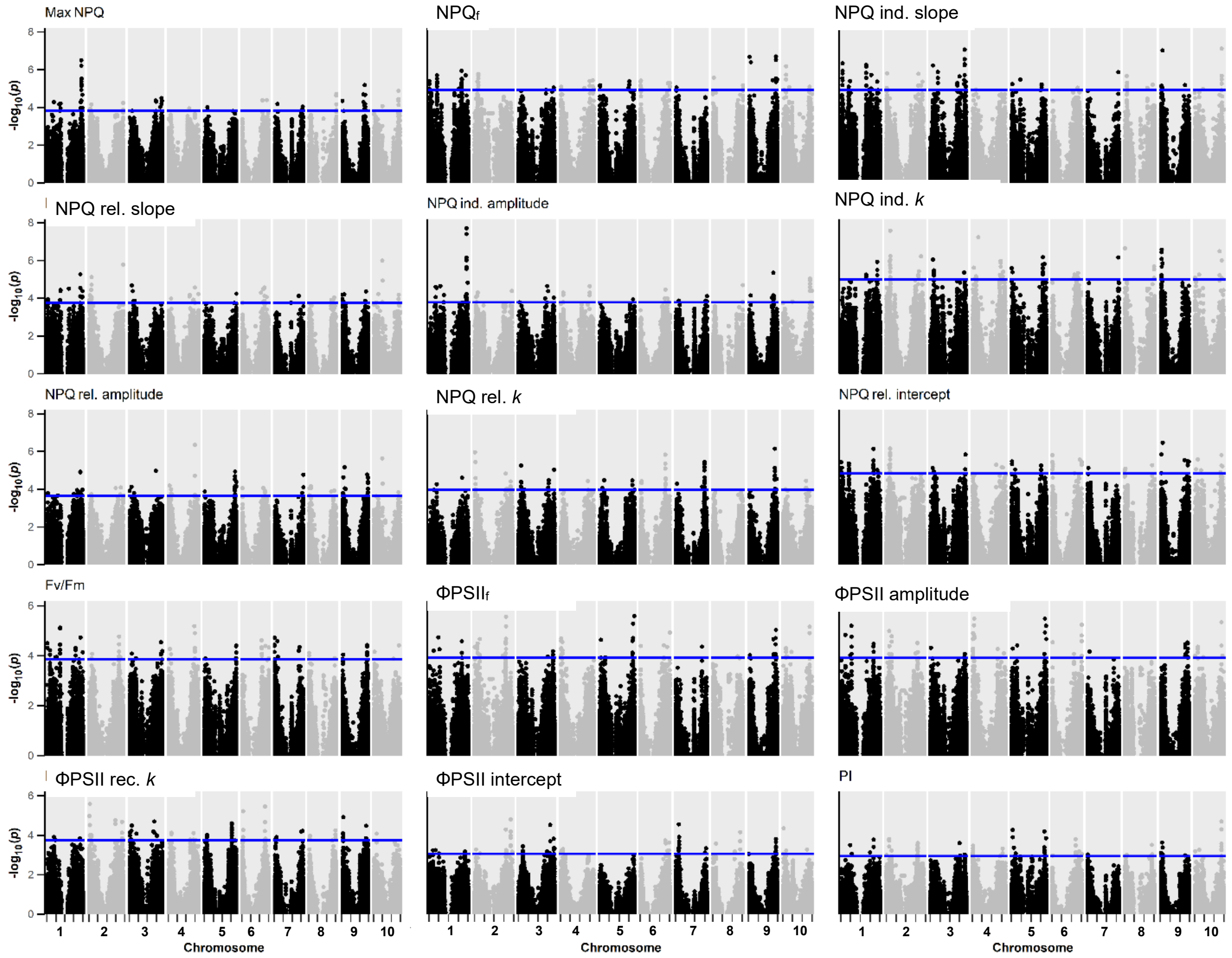

Figure S3: Chromosome mapping (physical location) for single nucleotide polymorphisms (SNPs) associated with 2017 genome-wide association study (GWAS) non-photochemical quenching traits. Blue line indicates threshold of SNPs in top 0.05% by -log$_{10}$ *p*-value. 65% of SNPs below *p*-value of 2.5 have been randomly removed from each GWAS plot to reduce image size. NPQf: Final dark time point NPQ value. Phi2: Photosystem II quantum yield. Phi2f: Final dark time point Phi2. PI: Photoprotection index.

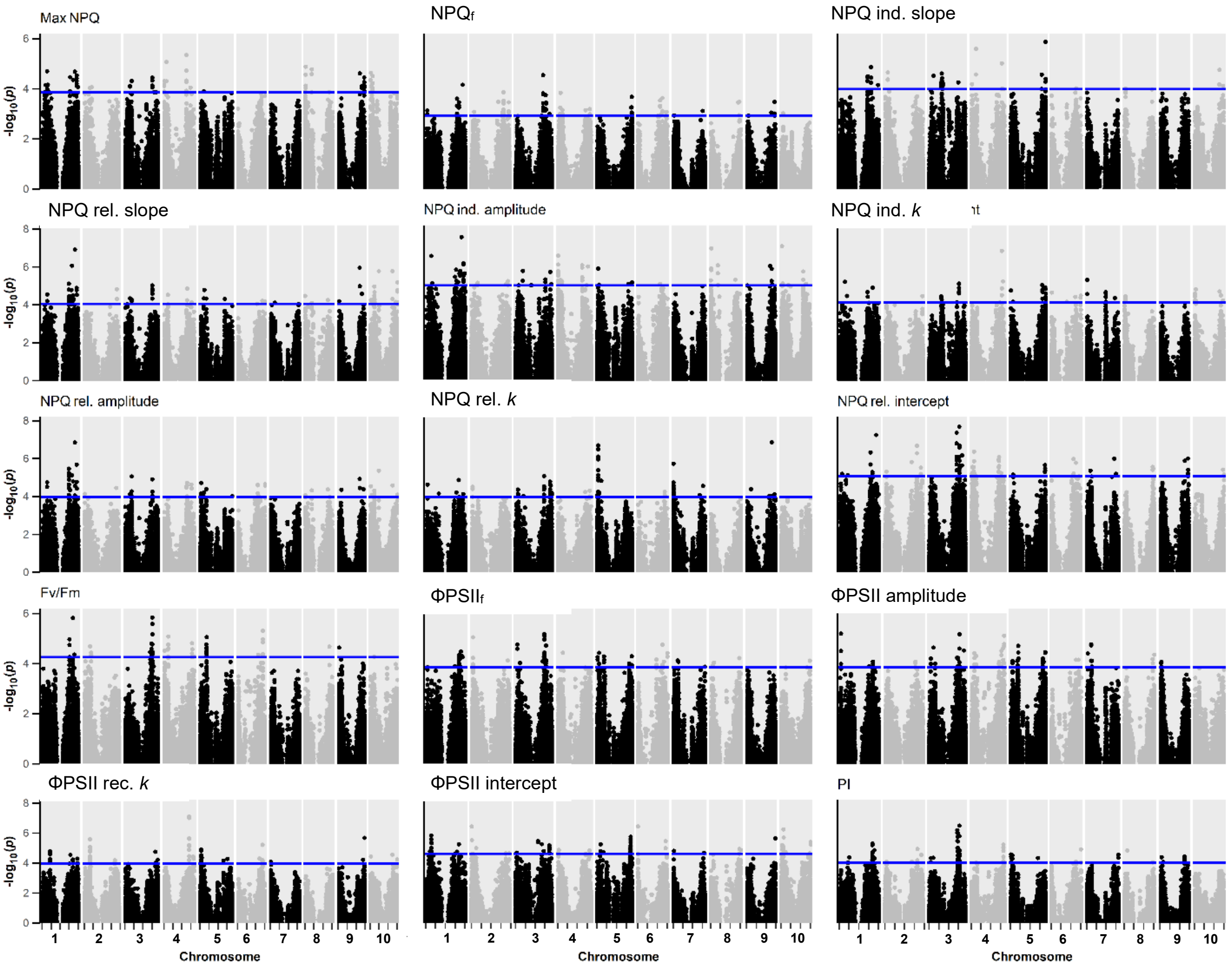

Figure S4: Chromosome mapping (physical location) for single nucleotide polymorphisms (SNPs) associated with 2019 genome-wide association study (GWAS) non-photochemical quenching traits. Blue line indicates threshold of SNPs in top 0.05% by $-\log_{10}$ *p*-value. 65% of SNPs below *p*-value of 2.5 have been randomly removed from each GWAS plot to reduce image size. NPQf: Final dark time point NPQ value. Phi2: Photosystem II quantum yield. Phi2f: Final dark time point Phi2. PI: Photoprotection index.

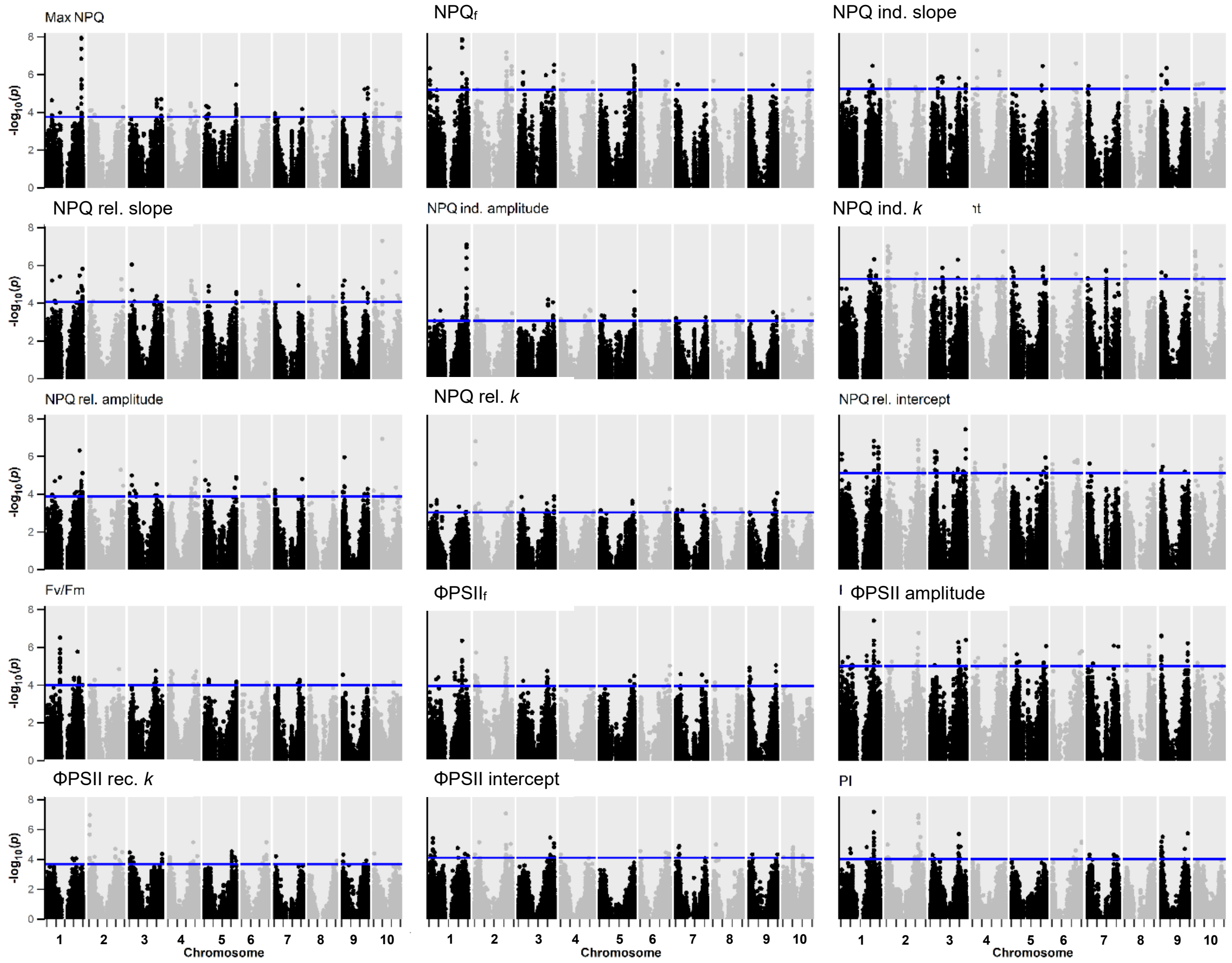

Figure S5: Chromosome mapping (physical location) for single nucleotide polymorphisms (SNPs) associated with joint genome-wide association study (GWAS) non-photochemical quenching traits. Blue line indicates threshold of SNPs in top 0.05% by $-\log_{10}$ *p*-value. 65% of SNPs below *p*-value of 2.5 have been randomly removed from each GWAS plot to reduce image size. NPQf: Final dark time point NPQ value. Phi2: Photosystem II quantum yield. Phi2f: Final dark time point Phi2. PI: Photoprotection index.

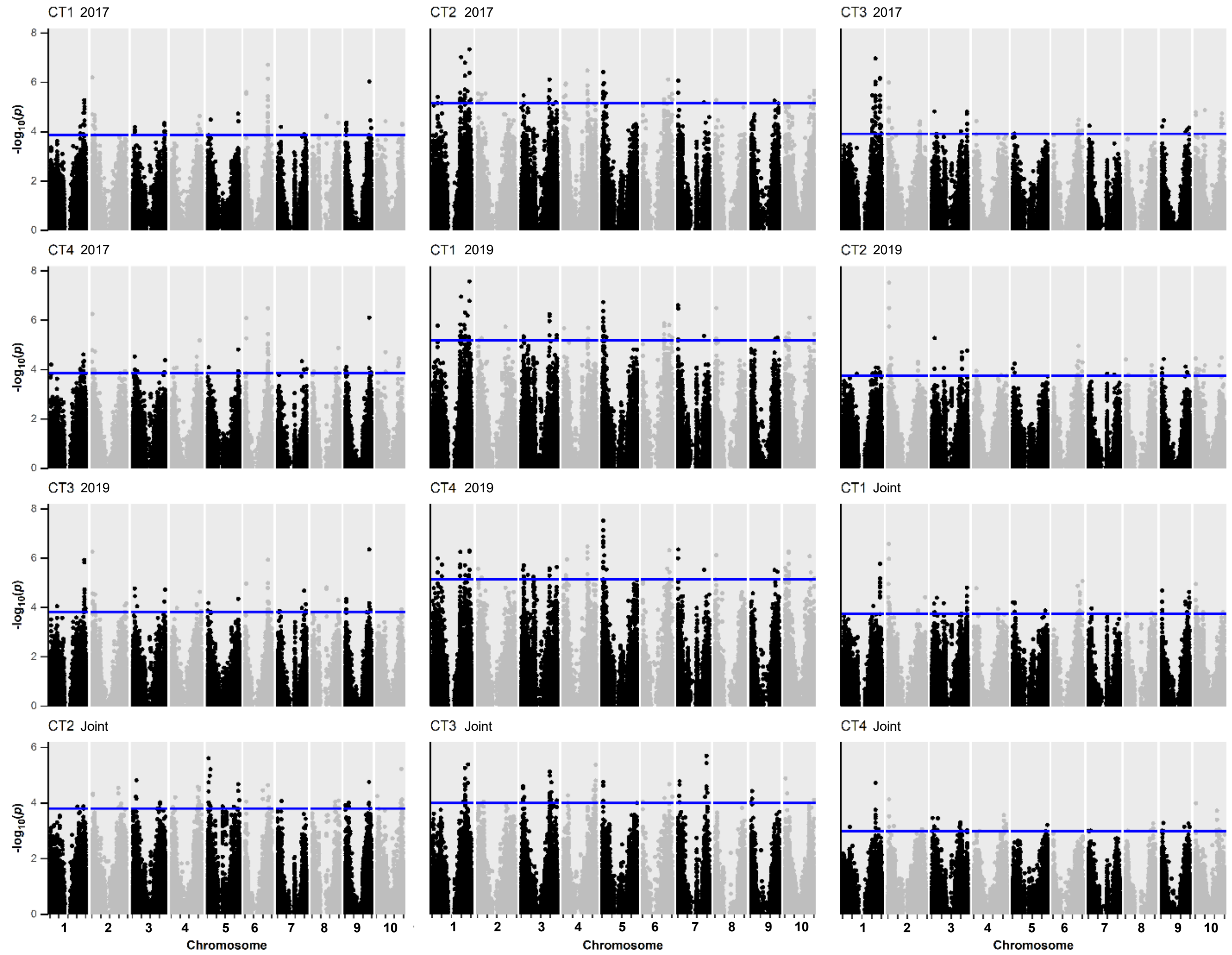

Figure S6: Chromosome mapping (physical location) for single nucleotide polymorphisms (SNPs) associated with combined-trait (CT) genome-wide association study (GWAS) non-photochemical quenching traits. Blue line indicates threshold of SNPs in top 0.05% by -$\log_{10}$ *p*-value. 65% of SNPs below *p*-value of 2.5 have been randomly removed from each GWAS plot to reduce image size. CT1: Max NPQ, NPQ induction amplitude, NPQ induction rate constant *k*, NPQ relaxation rate constant *k*. CT2: NPQ induction amplitude, NPQ induction *k*, NPQ relaxation rate constant *k*. CT3: Max NPQ, NPQ induction rate constant *k*, NPQ relaxation rate constant *k*. CT4: *PI*, *ΦPSII* recovery amplitude, *ΦPSII* recovery rate constant *k*.

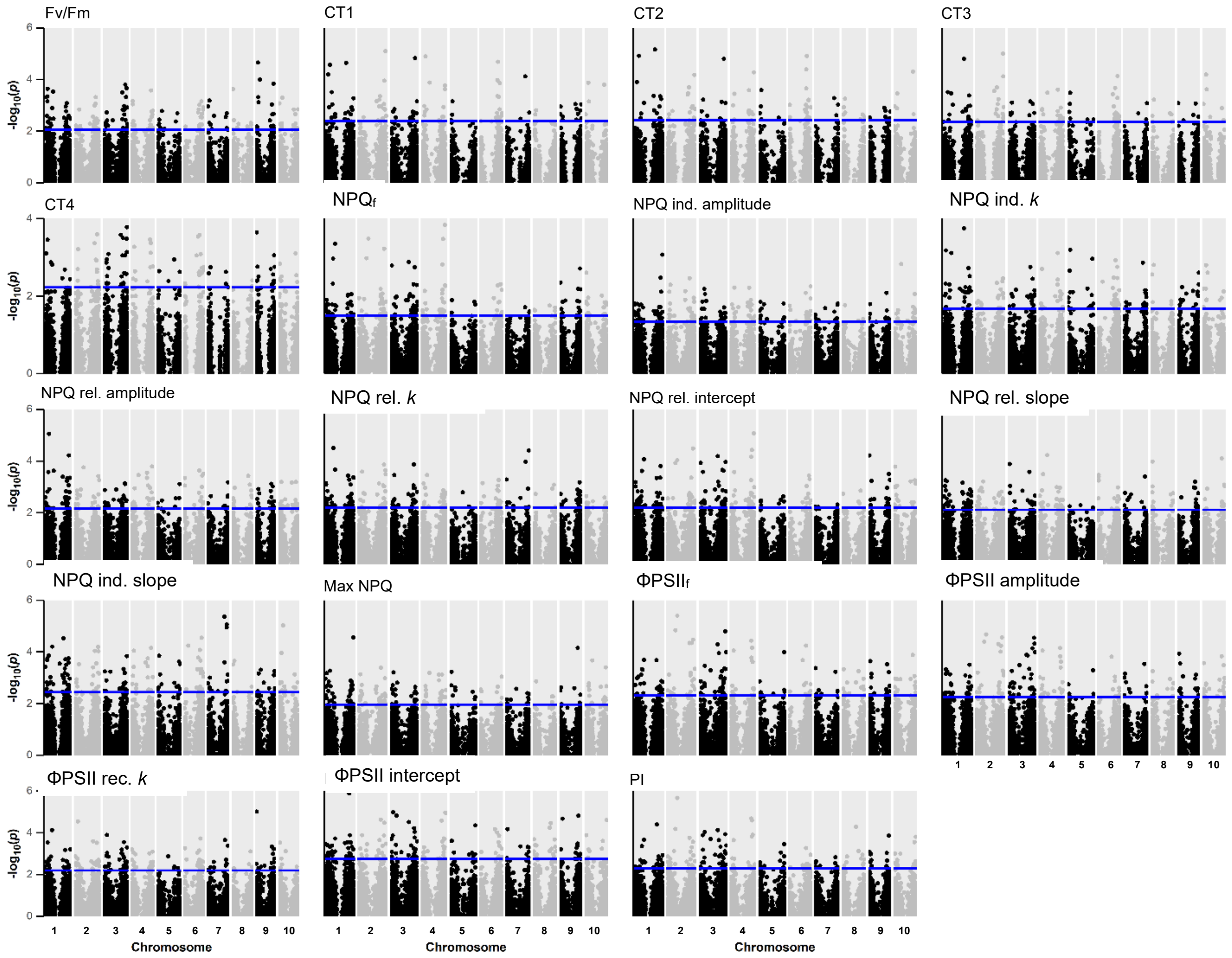

Figure S7: Chromosome mapping (physical location) for genes associated with 2017 third-leaf tissue transcriptome-wide association study (TWAS) non-photochemical quenching traits. Gene positions plotted as midpoint of each gene. Blue line indicates threshold of genes in top 1% by $-\log_{10}$ *p*-value. CT: Combined trait analysis. CT1: Max NPQ, NPQ induction amplitude, NPQ induction rate constant *k*, NPQ relaxation rate constant *k*. CT2: NPQ induction amplitude, NPQ induction *k*, NPQ relaxation rate constant *k*. CT3: Max NPQ, NPQ induction rate constant *k*, NPQ relaxation rate constant *k*. CT4: *PI*, *ΦPSII* recovery amplitude, *ΦPSII* recovery rate constant *k*. NPQf: Final dark time point NPQ value. Phi2: Photosystem II quantum yield. Phi2f: Final dark time point Phi2. PI: Photoprotection index.

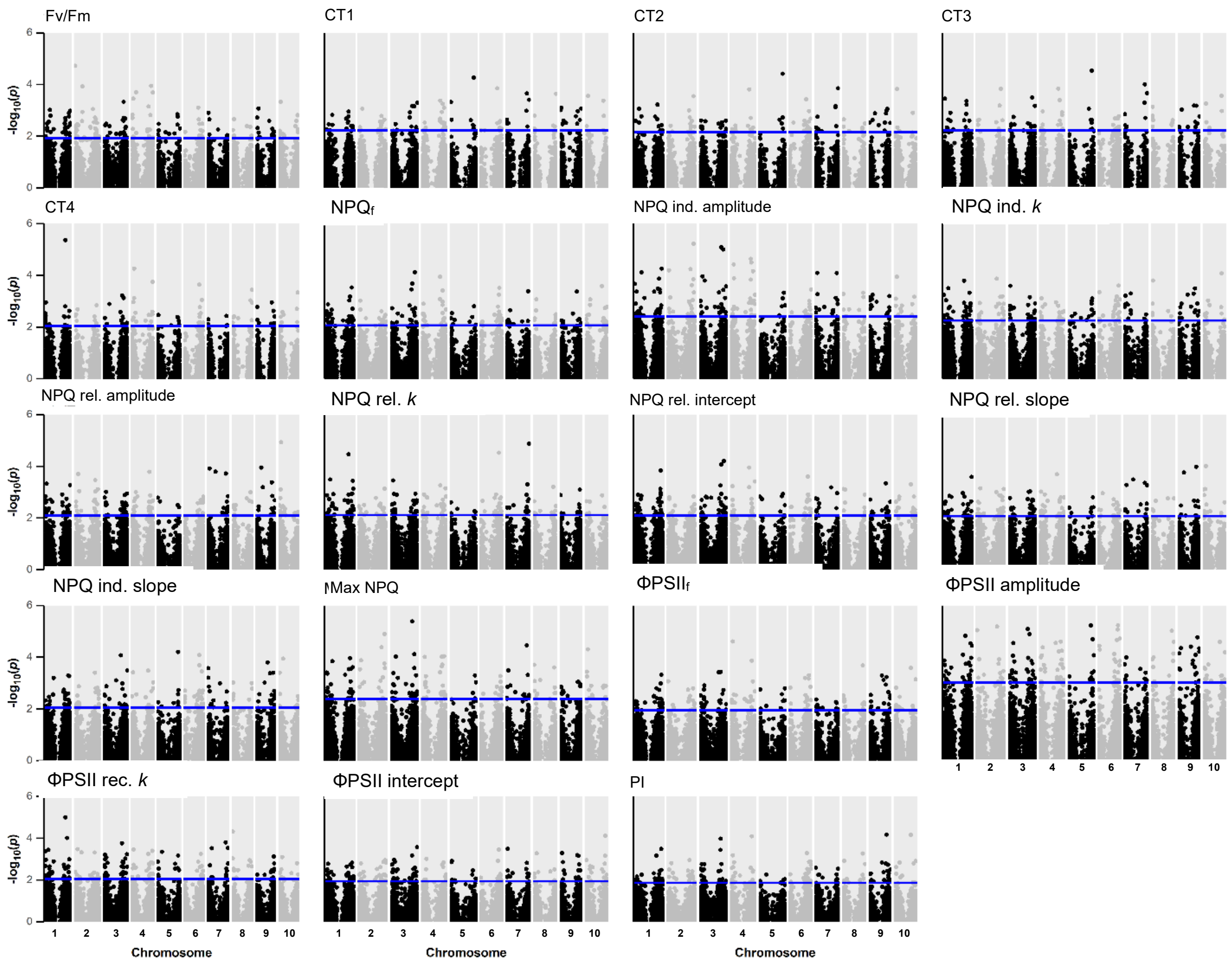

Figure S8: Chromosome mapping (physical location) for genes associated with 2019 third-leaf tissue transcriptome-wide association study (TWAS) non-photochemical quenching traits. Gene positions plotted as midpoint of each gene. Blue line indicates threshold of genes in top 1% by -$\log_{10}$ *p*-value. CT: Combined trait analysis. CT1: Max NPQ, NPQ induction amplitude, NPQ induction rate constant *k*, NPQ relaxation rate constant *k*. CT2: NPQ induction amplitude, NPQ induction *k*, NPQ relaxation rate constant *k*. CT3: Max NPQ, NPQ induction rate constant *k*, NPQ relaxation rate constant *k*. CT4: *PI*, *ΦPSII* recovery amplitude, *ΦPSII* recovery rate constant *k*. NPQf: Final dark time point NPQ value. Phi2: Photosystem II quantum yield. Phi2f: Final dark time point Phi2. PI: Photoprotection index.

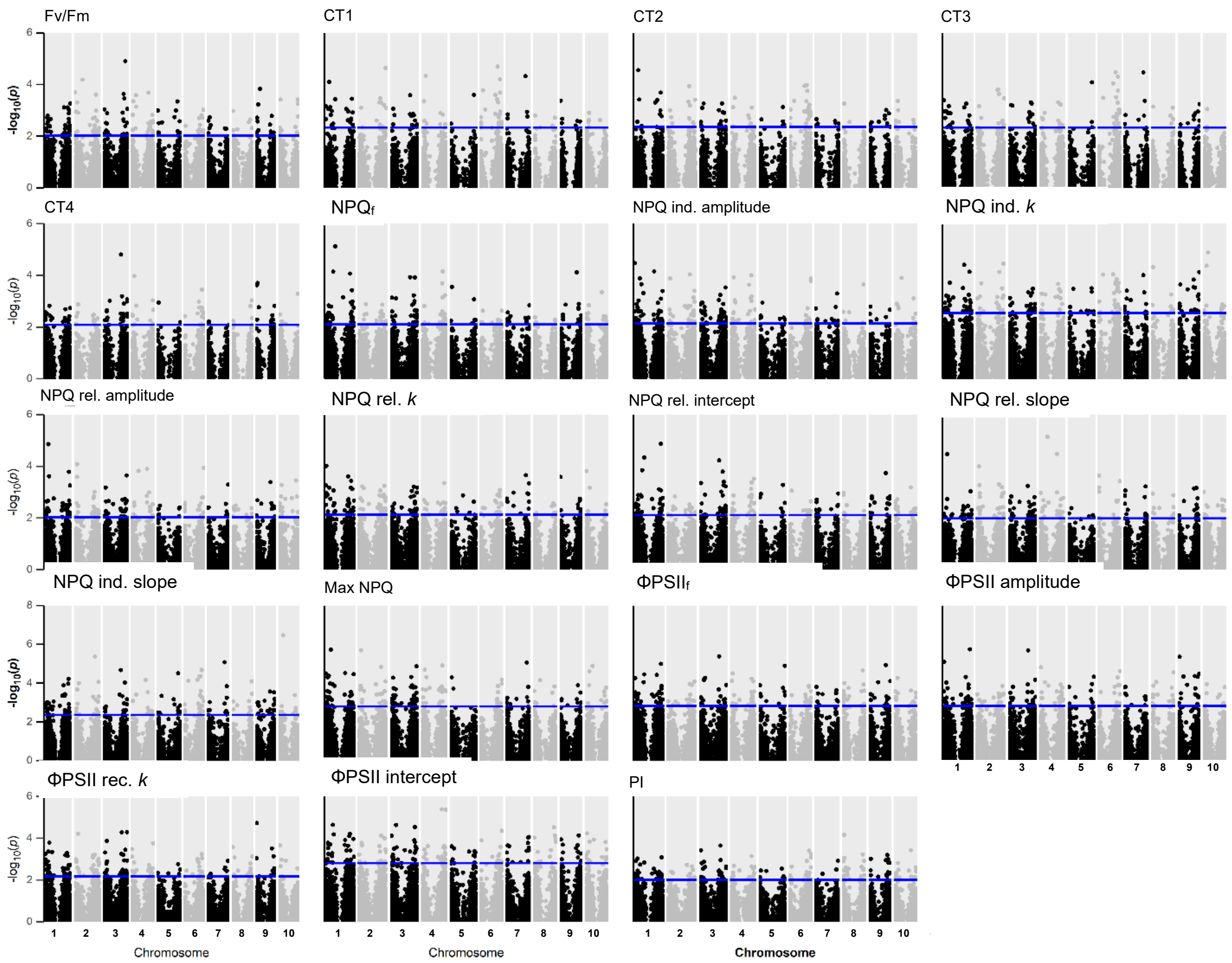

Figure S9: Chromosome mapping (physical location) for genes associated with joint analysis third-leaf tissue transcriptome-wide association study (TWAS) non-photochemical quenching traits. Gene positions plotted as midpoint of each gene. Blue line indicates threshold of genes in top 1% by -$\log_{10}$ *p*-value. CT: Combined trait analysis. CT1: Max NPQ, NPQ induction amplitude, NPQ induction rate constant *k*, NPQ relaxation rate constant *k*. CT2: NPQ induction amplitude, NPQ induction *k*, NPQ relaxation rate constant *k*. CT3: Max NPQ, NPQ induction rate constant *k*, NPQ relaxation rate constant *k*. CT4: *PI*, *ΦPSII* recovery amplitude, *ΦPSII* recovery rate constant *k*. NPQf: Final dark time point NPQ value. Phi2: Photosystem II quantum yield. Phi2f: Final dark time point Phi2. PI: Photoprotection index.

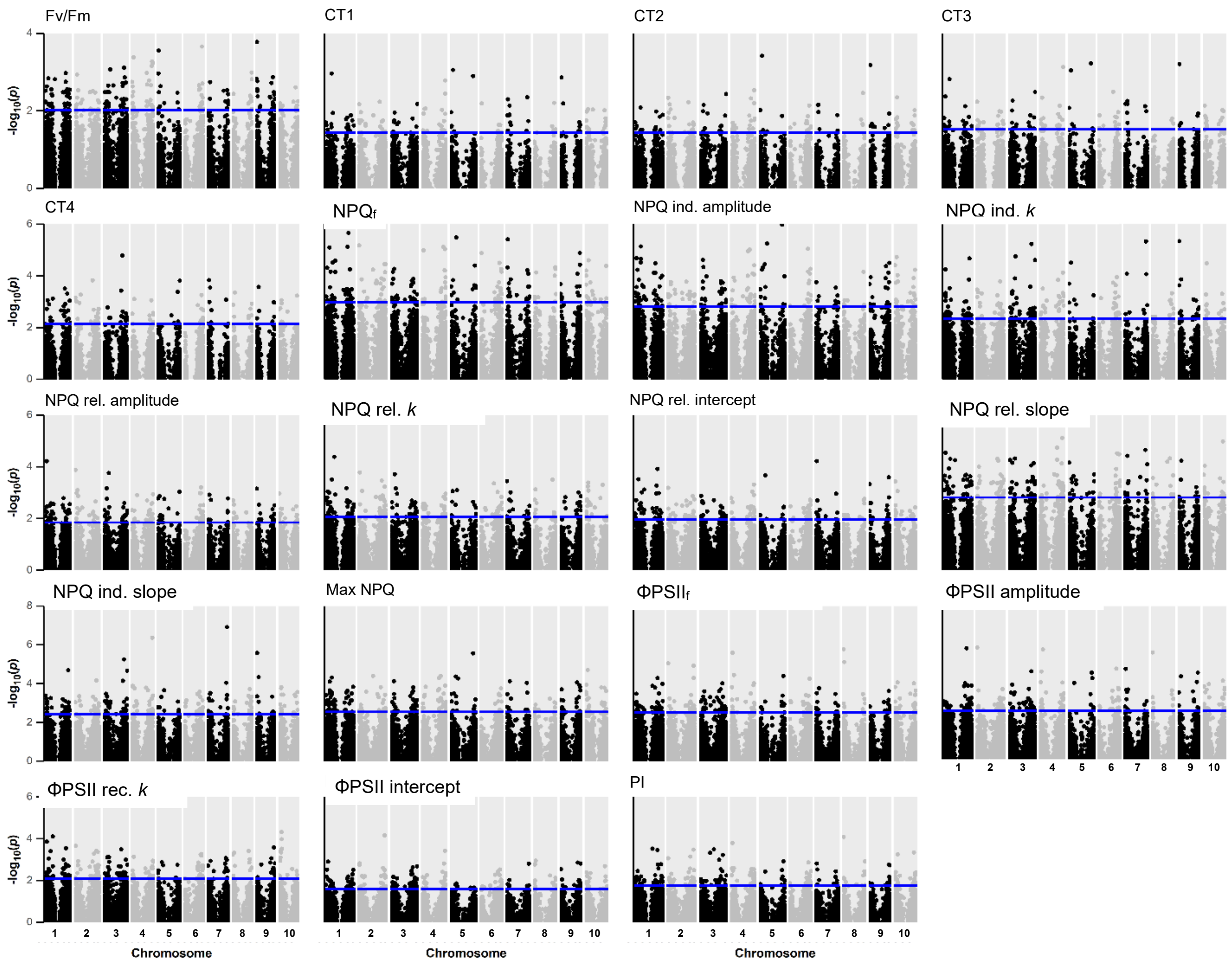

Figure S10: Chromosome mapping (physical location) for genes associated with 2017 growing point tissue transcriptome-wide association study (TWAS) non-photochemical quenching traits. Gene positions plotted as midpoint of each gene. Blue line indicates threshold of genes in top 1% by -$\log_{10}$ *p*-value. CT: Combined trait analysis. CT1: Max NPQ, NPQ induction amplitude, NPQ induction rate constant *k*, NPQ relaxation rate constant *k*. CT2: NPQ induction amplitude, NPQ induction *k*, NPQ relaxation rate constant *k*. CT3: Max NPQ, NPQ induction rate constant *k*, NPQ relaxation rate constant *k*. CT4: *PI*, *ΦPSII* recovery amplitude, *ΦPSII* recovery rate constant *k*. NPQf: Final dark time point NPQ value. Phi2: Photosystem II quantum yield. Phi2f: Final dark time point Phi2. PI: Photoprotection index.

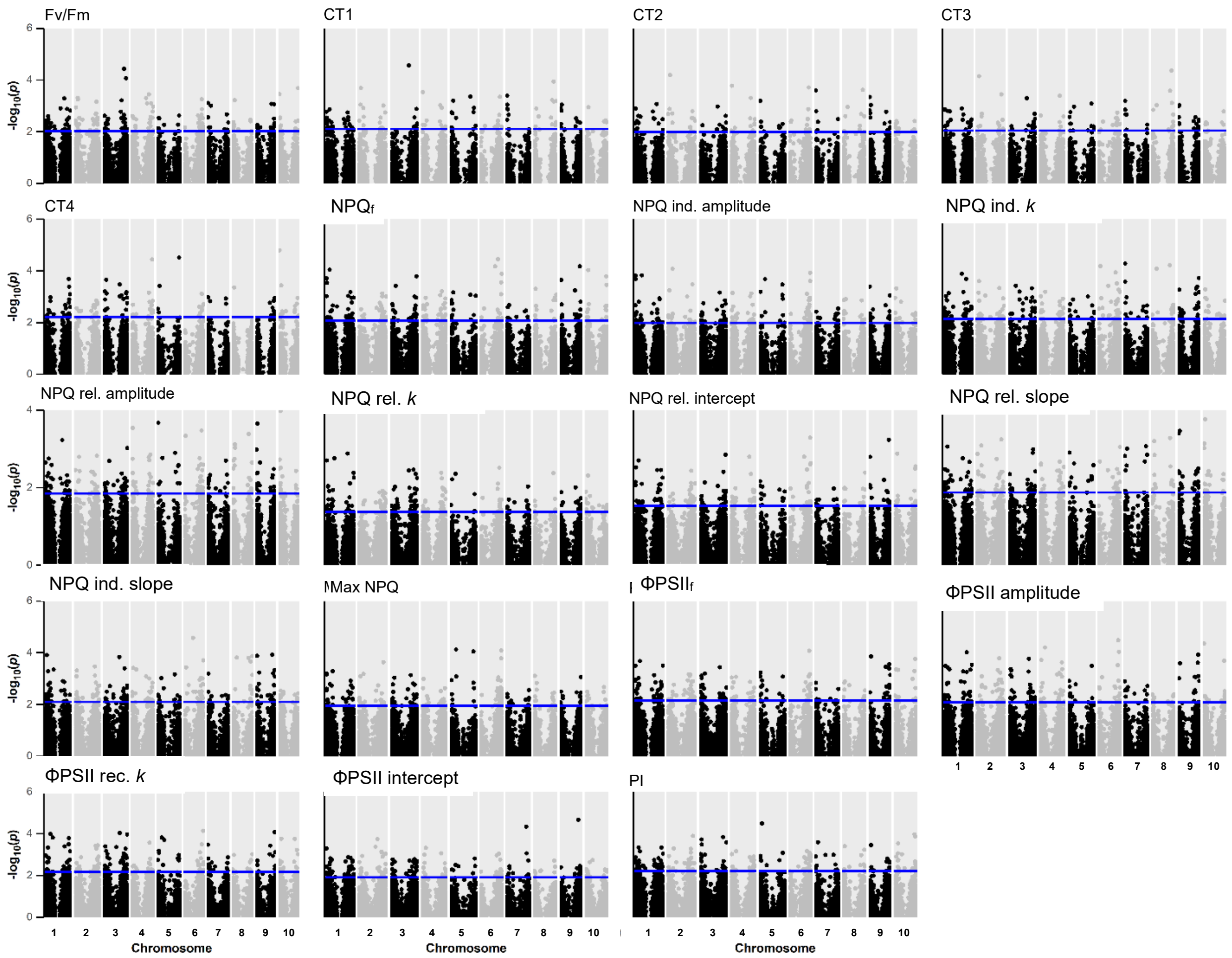

Figure S11: Chromosome mapping (physical location) for genes associated with 2019 growing point tissue transcriptome-wide association study (TWAS) non-photochemical quenching traits. Gene positions plotted as midpoint of each gene. Blue line indicates threshold of genes in top 1% by -$\log_{10}$ *p*-value. CT: Combined trait analysis. CT1: Max NPQ, NPQ induction amplitude, NPQ induction rate constant *k*, NPQ relaxation rate constant *k*. CT2: NPQ induction amplitude, NPQ induction *k*, NPQ relaxation rate constant *k*. CT3: Max NPQ, NPQ induction rate constant *k*, NPQ relaxation rate constant *k*. CT4: *PI*, *ΦPSII* recovery amplitude, *ΦPSII* recovery rate constant *k*. NPQf: Final dark time point NPQ value. Phi2: Photosystem II quantum yield. Phi2f: Final dark time point Phi2. PI: Photoprotection index.

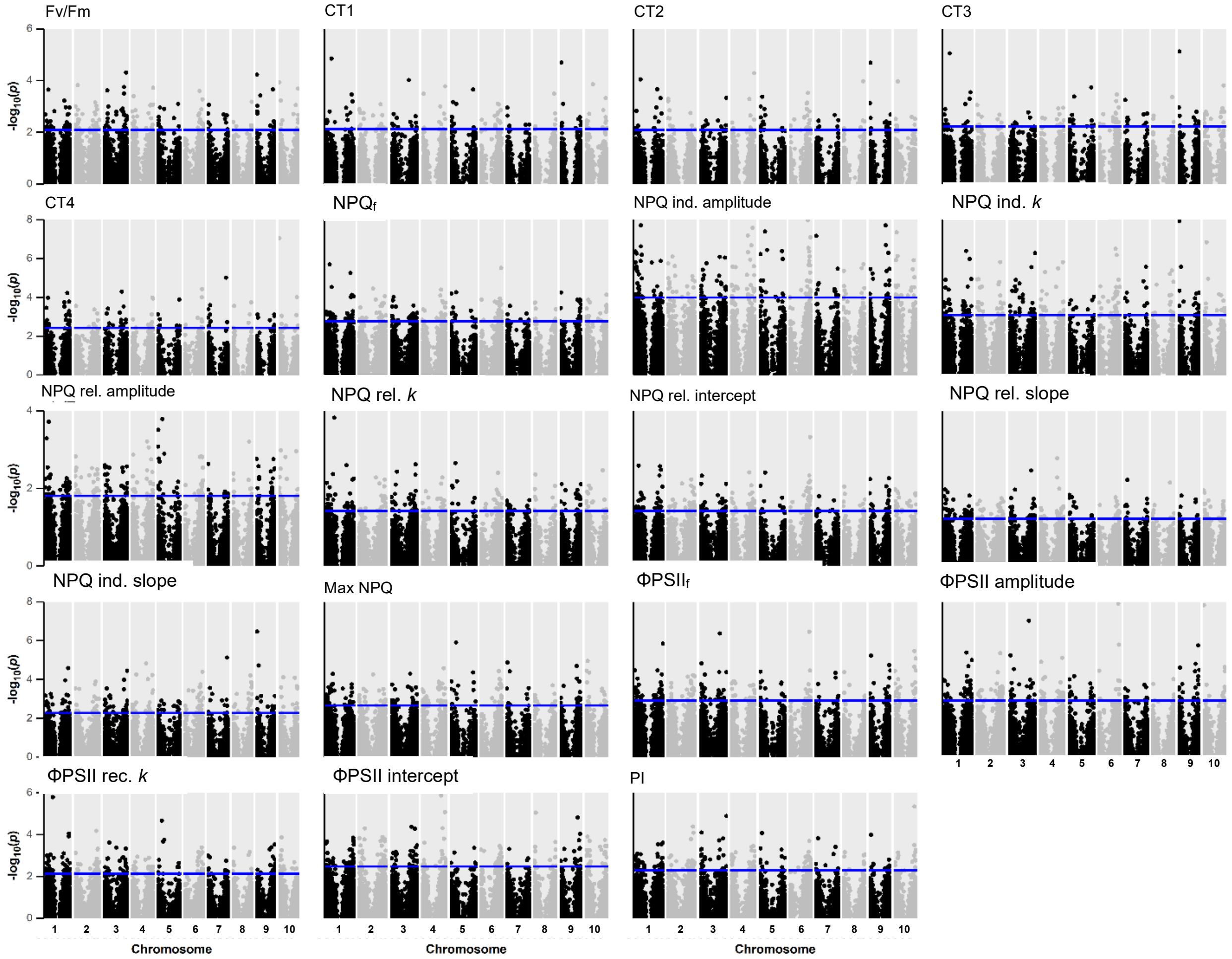

Figure S12: Chromosome mapping (physical location) for genes associated with joint analysis growing point tissue transcriptome-wide association study (TWAS) non-photochemical quenching traits. Gene positions plotted as midpoint of each gene. Blue line indicates threshold of genes in top 1% by -log$_{10}$ *p*-value. CT: Combined trait analysis. CT1: Max NPQ, NPQ induction amplitude, NPQ induction rate constant *k*, NPQ relaxation rate constant *k*. CT2: NPQ induction amplitude, NPQ induction *k*, NPQ relaxation rate constant *k*. CT3: Max NPQ, NPQ induction rate constant *k*, NPQ relaxation rate constant *k*. CT4: *PI*, *ΦPSII* recovery amplitude, *ΦPSII* recovery rate constant *k*. NPQf: Final dark time point NPQ value. Phi2: Photosystem II quantum yield. Phi2f: Final dark time point Phi2. PI: Photoprotection index.

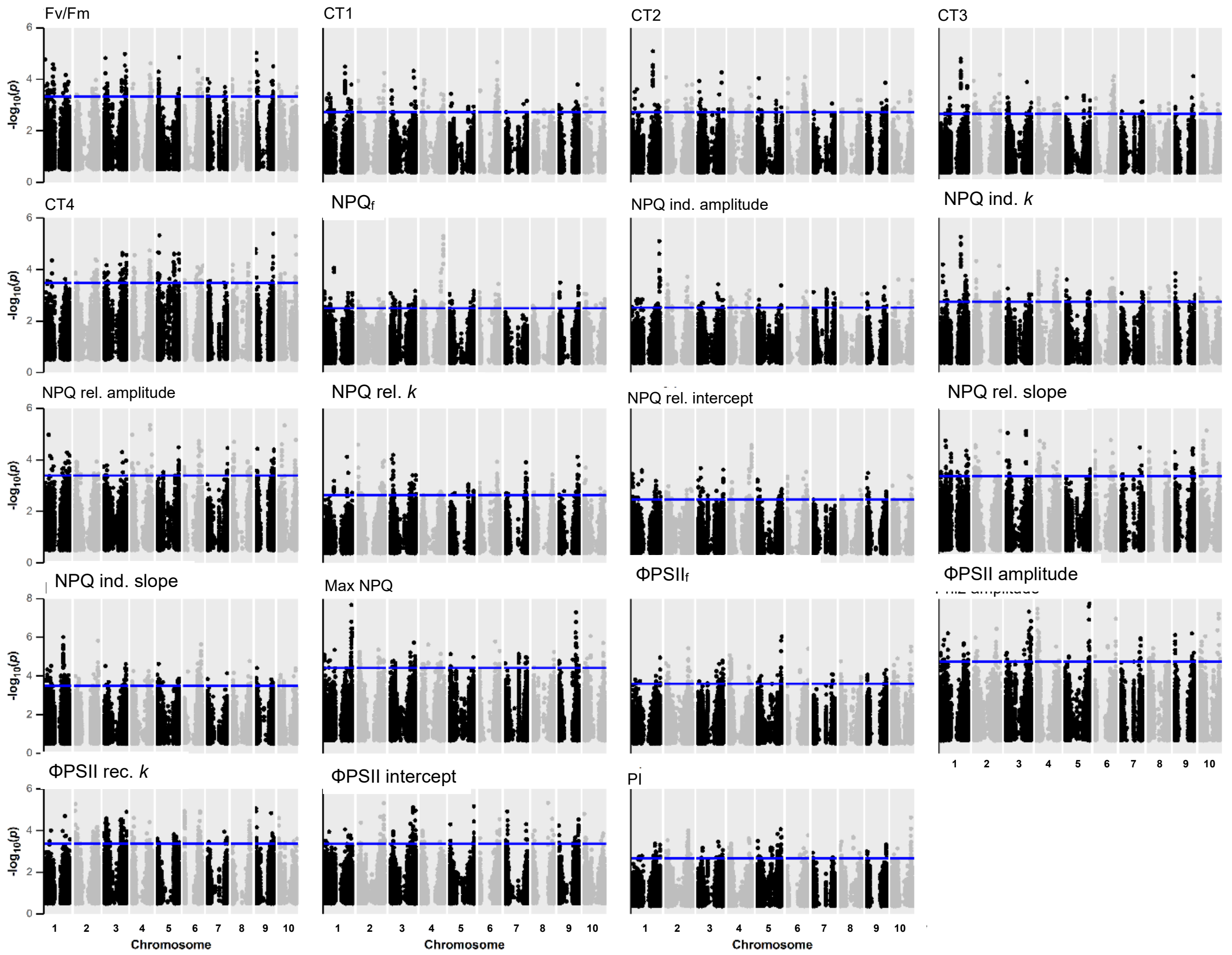

Figure S13: Chromosome mapping (physical location) for genes associated with 2017 third-leaf tissue Fisher's combined test (FCT) non-photochemical quenching traits. Gene positions plotted as midpoint of each gene. Blue line indicates threshold of genes in top 1% by -log$_{10}$ *p*-value. CT: Combined trait analysis. CT1: Max NPQ, NPQ induction amplitude, NPQ induction rate constant *k*, NPQ relaxation rate constant *k*. CT2: NPQ induction amplitude, NPQ induction *k*, NPQ relaxation rate constant *k*. CT3: Max NPQ, NPQ induction rate constant *k*, NPQ relaxation rate constant *k*. CT4: *PI*, *ΦPSII* recovery amplitude, *ΦPSII* recovery rate constant *k*. NPQf: Final dark time point NPQ value. Phi2: Photosystem II quantum yield. Phi2f: Final dark time point Phi2. PI: Photoprotection index.

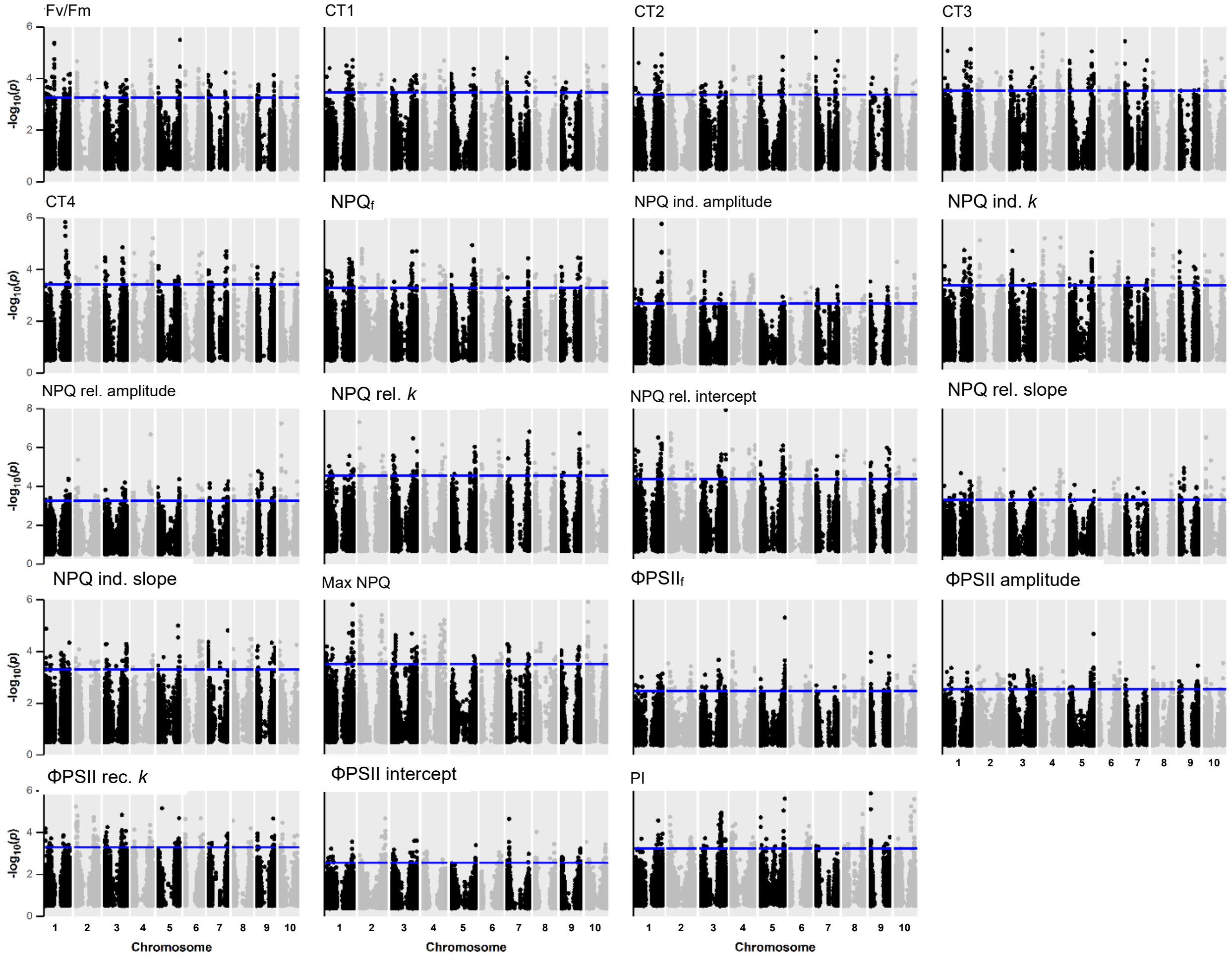

Figure S14: Chromosome mapping (physical location) for genes associated with 2019 third-leaf tissue Fisher's combined test (FCT) non-photochemical quenching traits. Gene positions plotted as midpoint of each gene. Blue line indicates threshold of genes in top 1% by $-\log_{10}$ *p*-value. CT: Combined trait analysis. CT1: Max NPQ, NPQ induction amplitude, NPQ induction rate constant *k*, NPQ relaxation rate constant *k*. CT2: NPQ induction amplitude, NPQ induction *k*, NPQ relaxation rate constant *k*. CT3: Max NPQ, NPQ induction rate constant *k*, NPQ relaxation rate constant *k*. CT4: *PI*, *ΦPSII* recovery amplitude, *ΦPSII* recovery rate constant *k*. NPQf: Final dark time point NPQ value. Phi2: Photosystem II quantum yield. Phi2f: Final dark time point Phi2. PI: Photoprotection index.

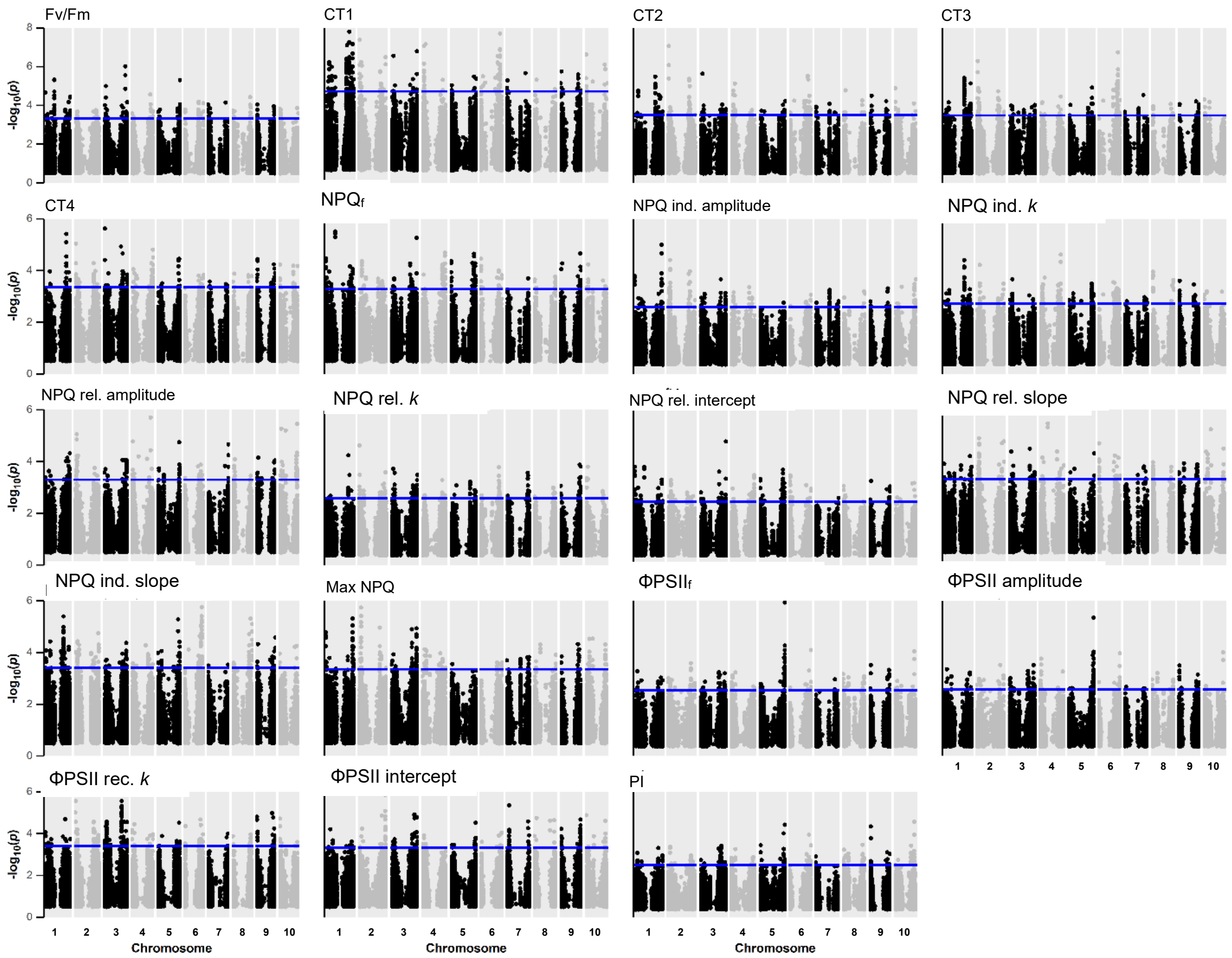

Figure S15: Chromosome mapping (physical location) for genes associated with joint analysis third-leaf tissue Fisher's combined test (FCT) non-photochemical quenching traits. Gene positions plotted as midpoint of each gene. Blue line indicates threshold of genes in top 1% by -log$_{10}$ *p*-value. CT: Combined trait analysis. CT1: Max NPQ, NPQ induction amplitude, NPQ induction rate constant *k*, NPQ relaxation rate constant *k*. CT2: NPQ induction amplitude, NPQ induction *k*, NPQ relaxation rate constant *k*. CT3: Max NPQ, NPQ induction rate constant *k*, NPQ relaxation rate constant *k*. CT4: *PI*, *ΦPSII* recovery amplitude, *ΦPSII* recovery rate constant *k*. NPQf: Final dark time point NPQ value. Phi2: Photosystem II quantum yield. Phi2f: Final dark time point Phi2. PI: Photoprotection index.

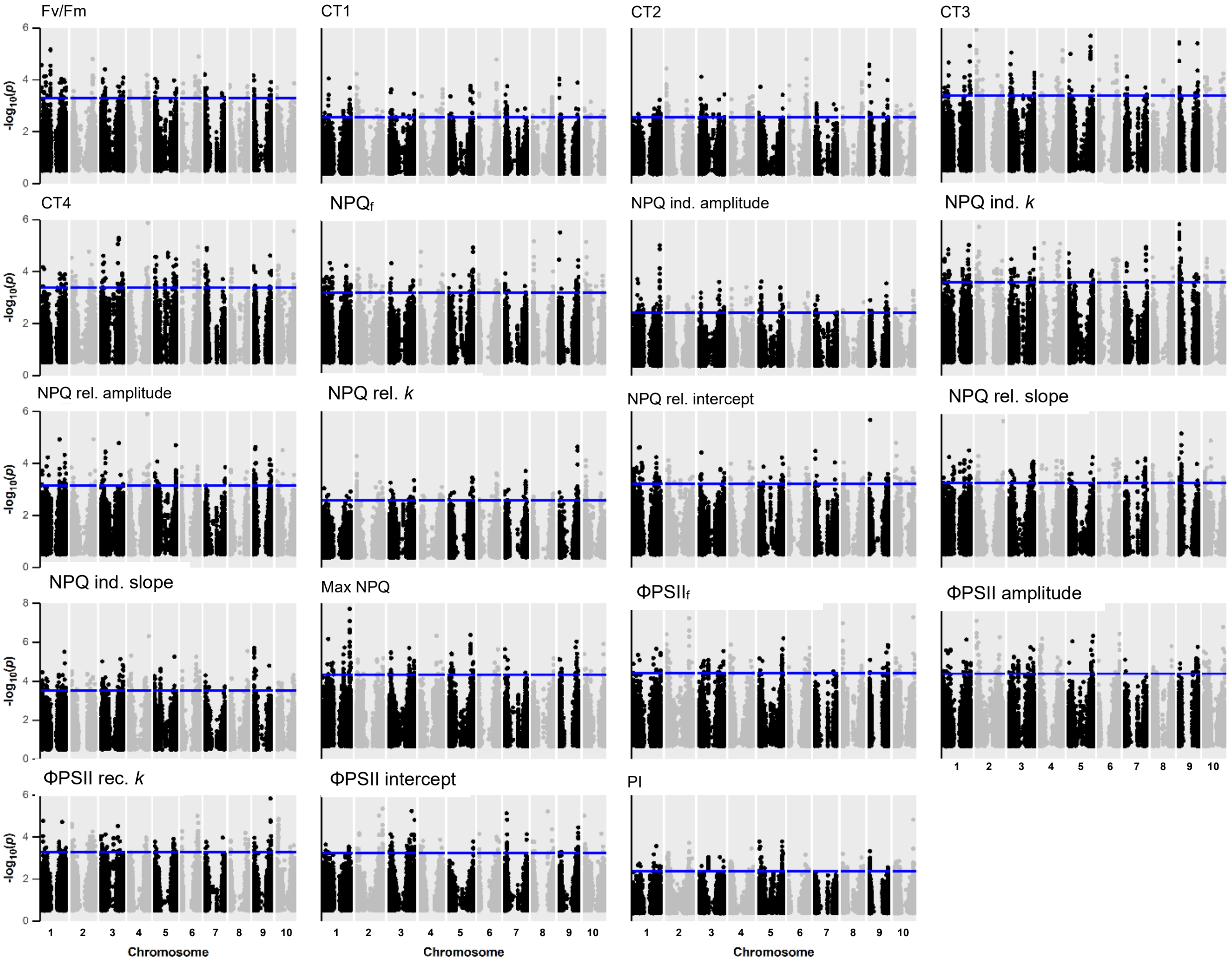

Figure S16: Chromosome mapping (physical location) for genes associated with 2017 growing point tissue Fisher's combined test (FCT) non-photochemical quenching traits. Gene positions plotted as midpoint of each gene. Blue line indicates threshold of genes in top 1% by -log$_{10}$ *p*-value. CT: Combined trait analysis. CT1: Max NPQ, NPQ induction amplitude, NPQ induction rate constant *k*, NPQ relaxation rate constant *k*. CT2: NPQ induction amplitude, NPQ induction *k*, NPQ relaxation rate constant *k*. CT3: Max NPQ, NPQ induction rate constant *k*, NPQ relaxation rate constant *k*. CT4: *PI*, *ΦPSII* recovery amplitude, *ΦPSII* recovery rate constant *k*. NPQf: Final dark time point NPQ value. Phi2: Photosystem II quantum yield. Phi2f: Final dark time point Phi2. PI: Photoprotection index.

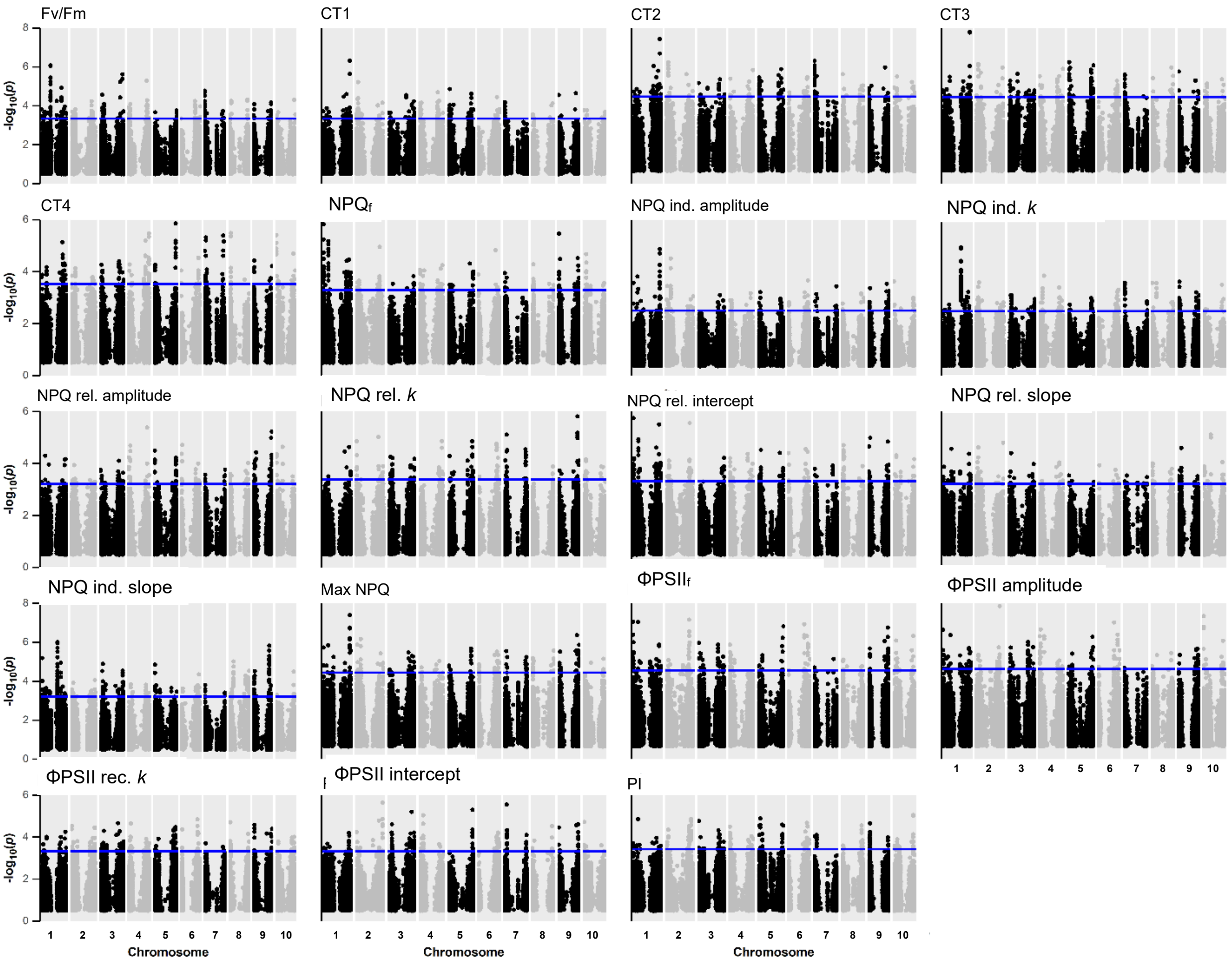

Figure S17: Chromosome mapping (physical location) for genes associated with 2019 growing point tissue Fisher's combined test (FCT) non-photochemical quenching traits. Gene positions plotted as midpoint of each gene. Blue line indicates threshold of genes in top 1% by $-\log_{10}$ *p*-value. CT: Combined trait analysis. CT1: Max NPQ, NPQ induction amplitude, NPQ induction rate constant *k*, NPQ relaxation rate constant *k*. CT2: NPQ induction amplitude, NPQ induction *k*, NPQ relaxation rate constant *k*. CT3: Max NPQ, NPQ induction rate constant *k*, NPQ relaxation rate constant *k*. CT4: *PI*, *ΦPSII* recovery amplitude, *ΦPSII* recovery rate constant *k*. NPQf: Final dark time point NPQ value. Phi2: Photosystem II quantum yield. Phi2f: Final dark time point Phi2. PI: Photoprotection index.

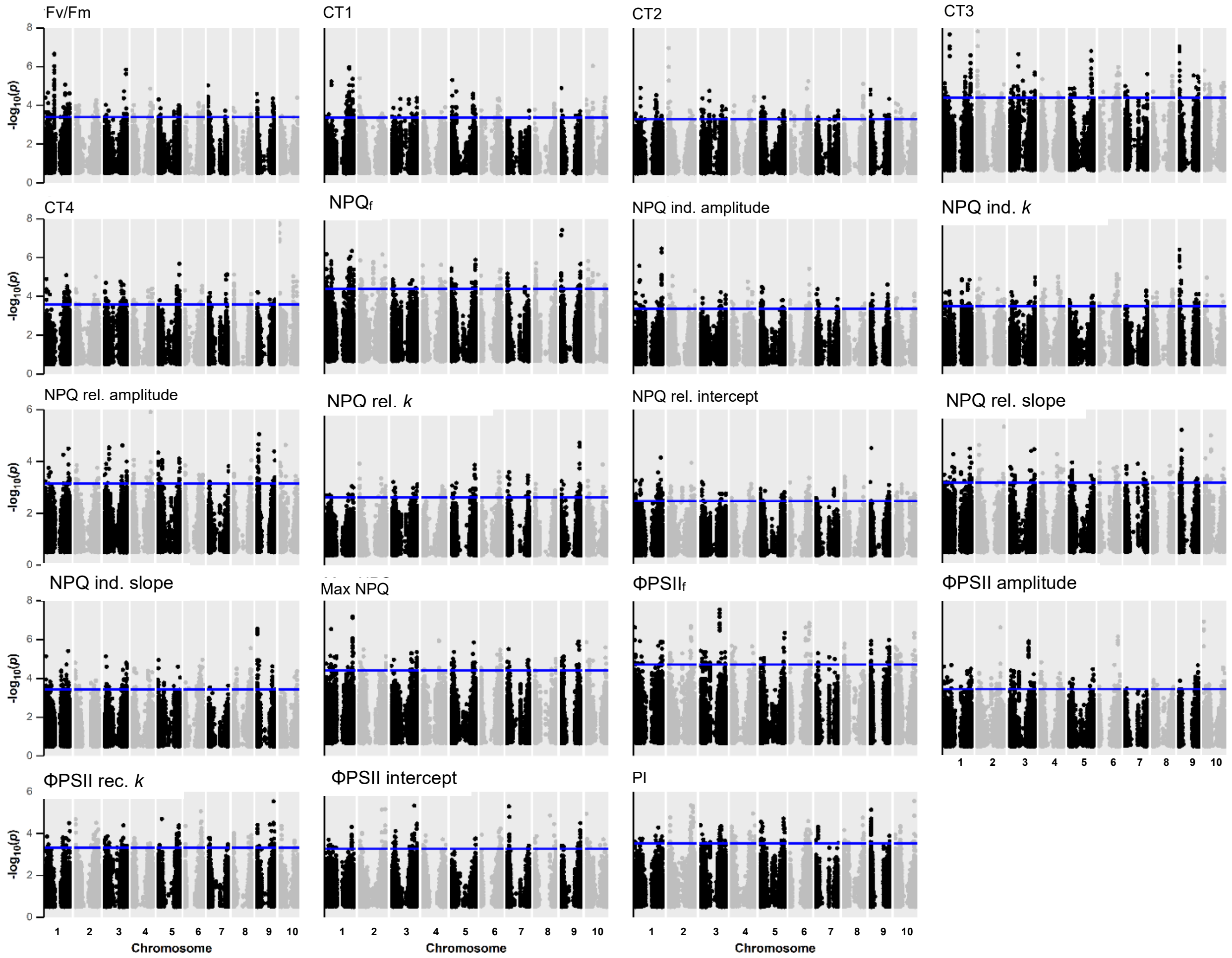

Figure S18: Chromosome mapping (physical location) for genes associated with joint analysis growing point tissue Fisher's combined test (FCT) non-photochemical quenching traits. Gene positions plotted as midpoint of each gene. Blue line indicates threshold of genes in top 1% by -$\log_{10}$ *p*-value. CT: Combined trait analysis. CT1: Max NPQ, NPQ induction amplitude, NPQ induction rate constant *k*, NPQ relaxation rate constant *k*. CT2: NPQ induction amplitude, NPQ induction *k*, NPQ relaxation rate constant *k*. CT3: Max NPQ, NPQ induction rate constant *k*, NPQ relaxation rate constant *k*. CT4: *PI*, *ΦPSII* recovery amplitude, *ΦPSII* recovery rate constant *k*. NPQf: Final dark time point NPQ value. Phi2: Photosystem II quantum yield. Phi2f: Final dark time point Phi2. PI: Photoprotection index.

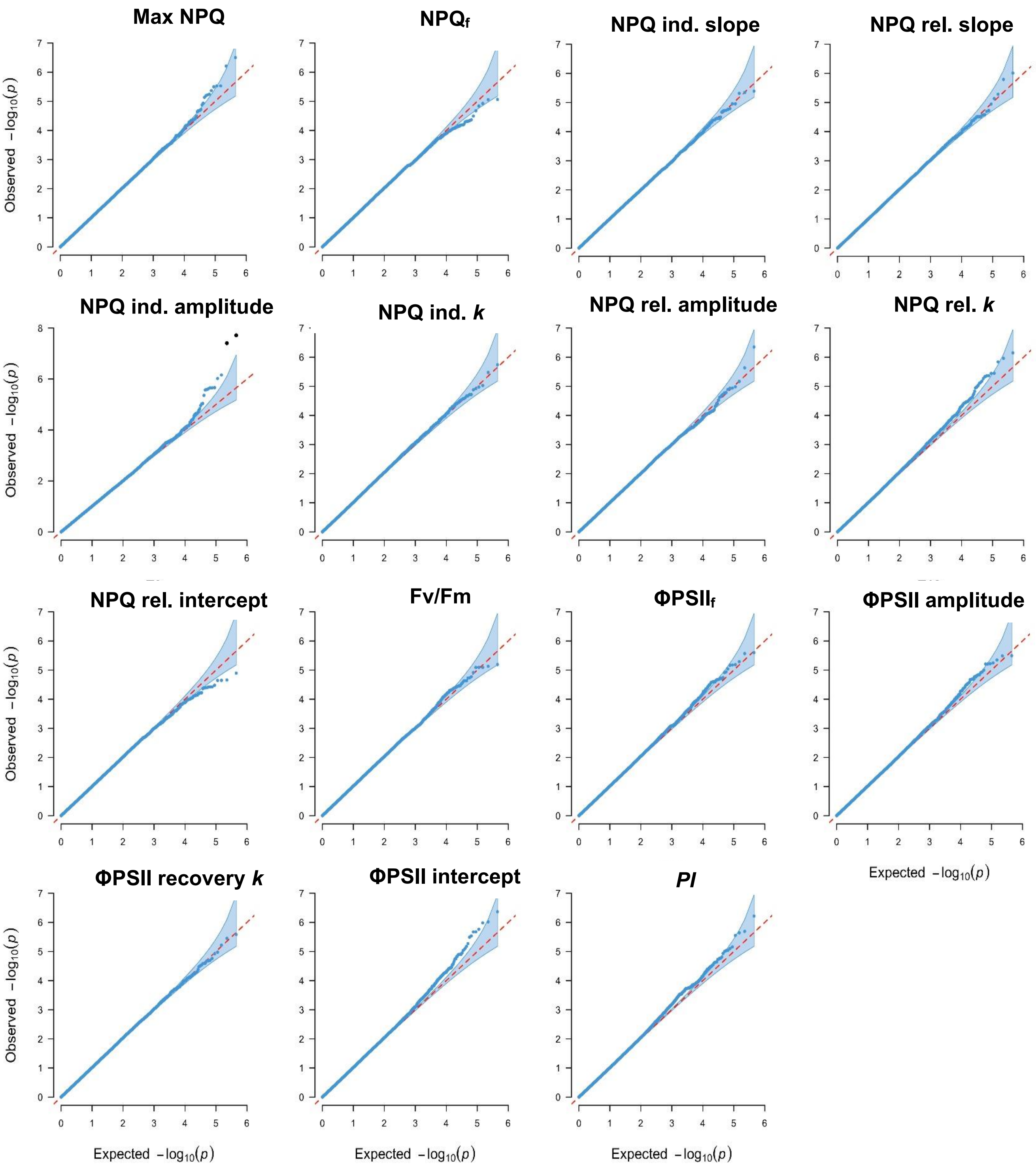

Figure S19: QQ plots for 2017 genome-wide association study (GWAS) single nucleotide polymorphism (SNPs) associated with non-photochemical quenching traits. Orange dashed line indicates 1:1 relationship between observed and expected -log10(*p*) values. Black points are SNPs with observed false discovery rate (FDR) adjusted *p*-values below 0.05. Shading is 95% confidence interval based on beta distribution (Yin et al., 2021).

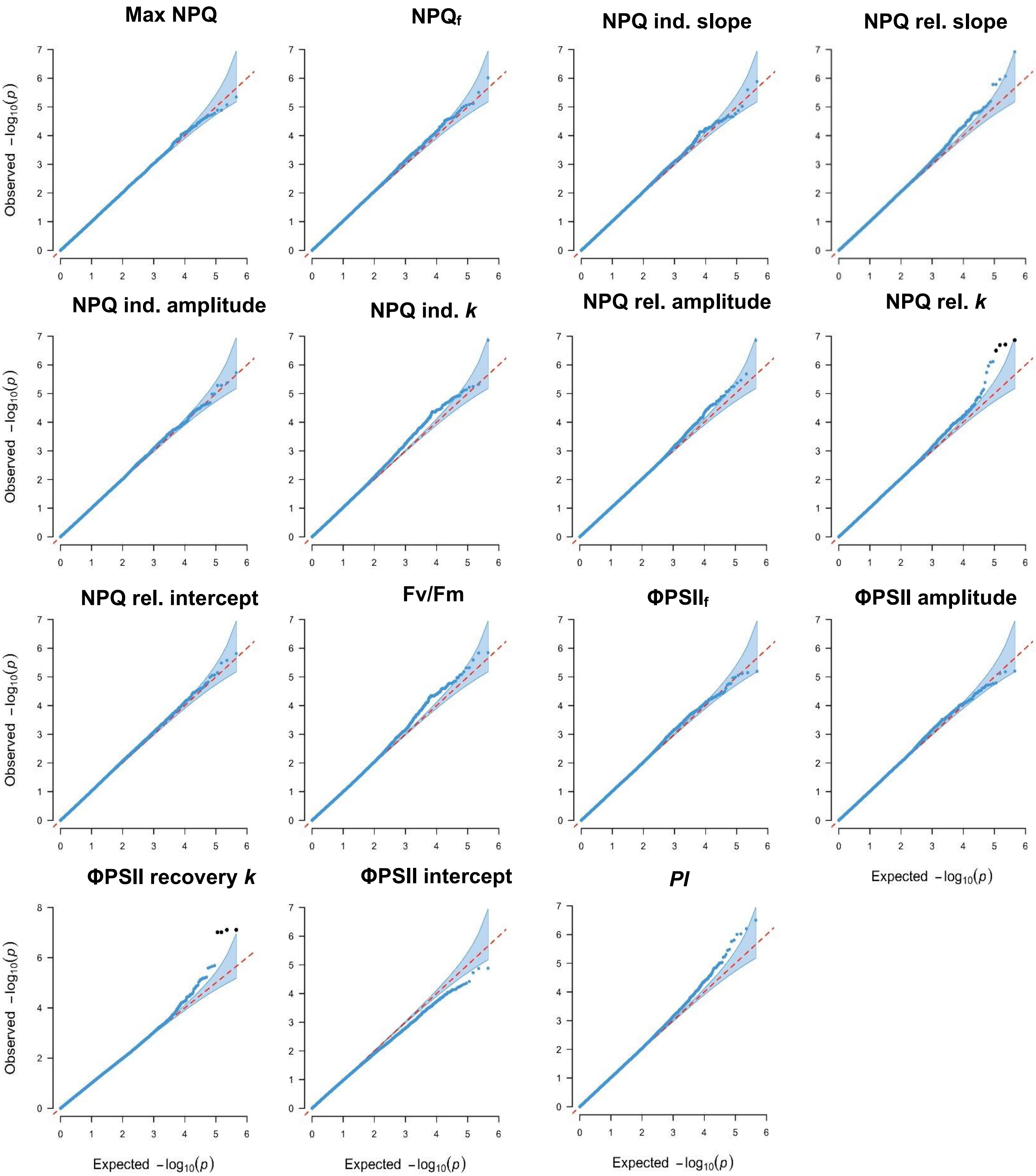

Figure S20: QQ plots for 2019 genome-wide association study (GWAS) single nucleotide polymorphism (SNPs) associated with non-photochemical quenching traits. Orange dashed line indicates 1:1 relationship between observed and expected -log10(*p*) values. Black points are SNPs with observed false discovery rate (FDR) adjusted *p*-values below 0.05. Shading is 95% confidence interval based on beta distribution (Yin et al., 2021).

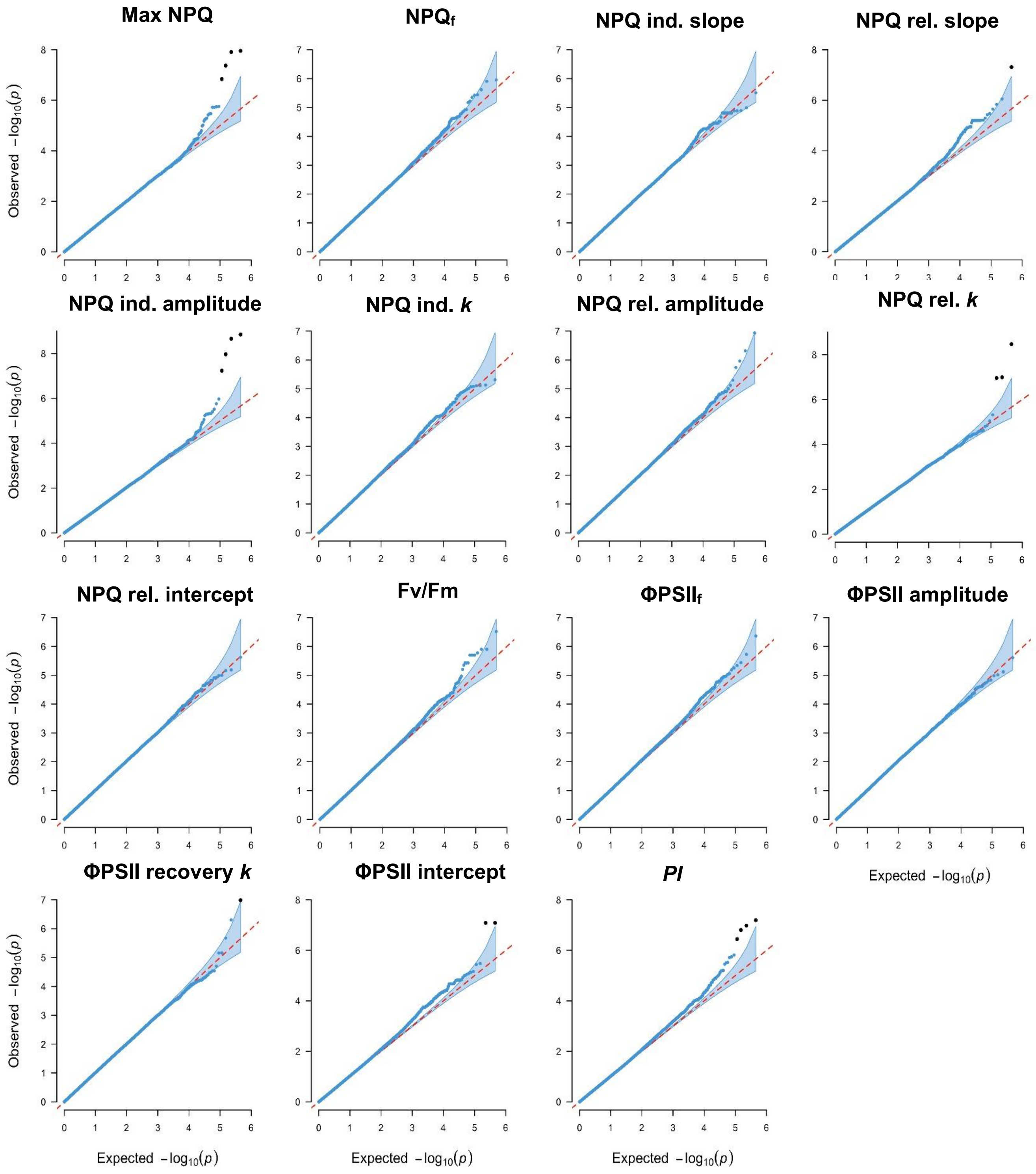

Figure S21: QQ plots for joint genome-wide association study (GWAS) single nucleotide polymorphism (SNPs) associated with non-photochemical quenching traits. Orange dashed line indicates 1:1 relationship between observed and expected -log10(*p*) values. Black points are SNPs with observed false discovery rate (FDR) adjusted *p*-values below 0.05. Shading is 95% confidence interval based on beta distribution (Yin et al., 2021).

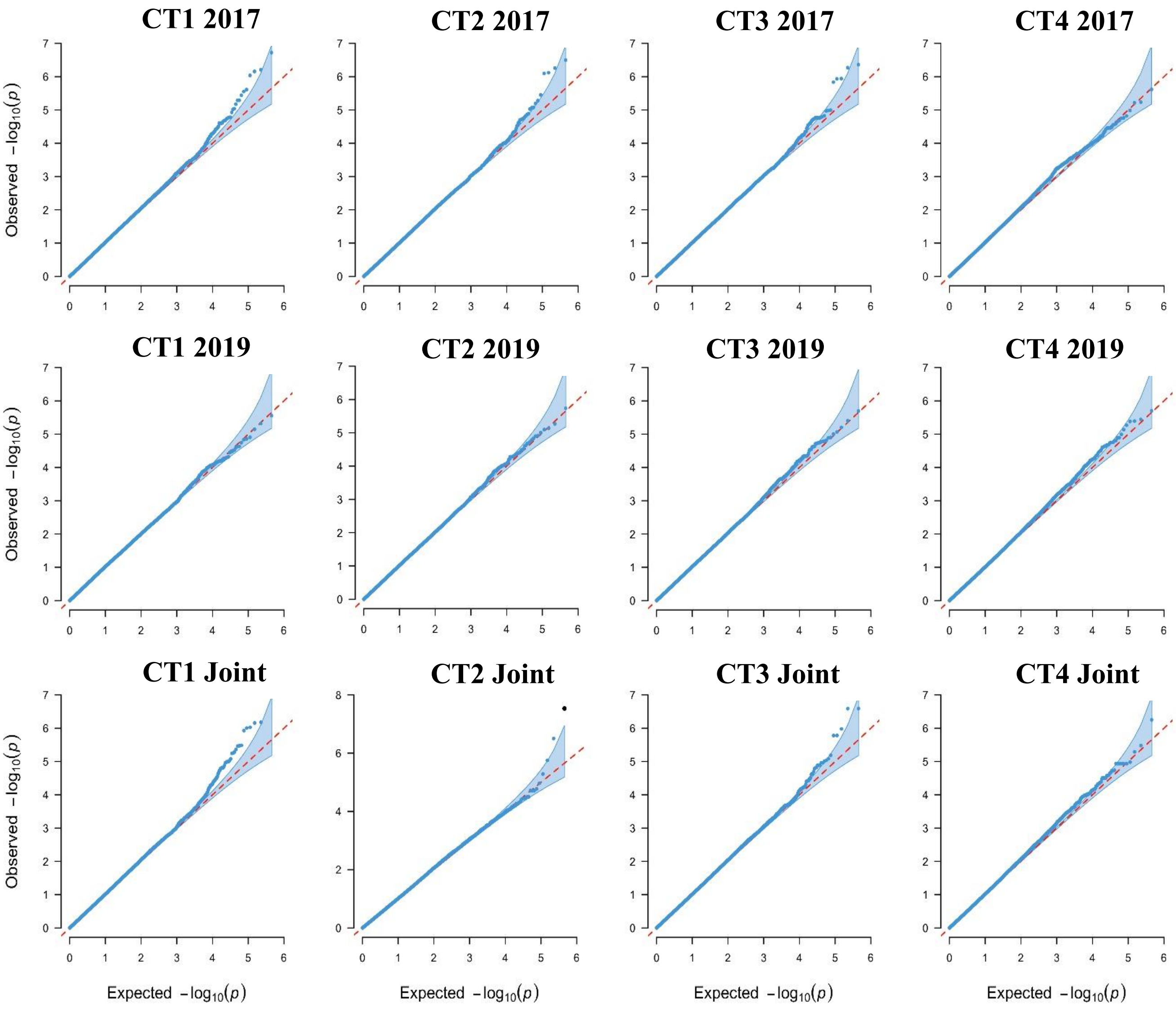

Figure S22: QQ plots for genome-wide association study (GWAS) single nucleotide polymorphism (SNPs) associated with combined non-photochemical quenching traits. Orange dashed line indicates 1:1 relationship between observed and expected -log10(*p*) values. Black points are SNPs with observed false discovery rate (FDR) adjusted *p*-values below 0.05. Shading is 95% confidence interval based on beta distribution (Yin et al., 2021). CT1: Max NPQ, NPQ induction amplitude, NPQ induction rate constant k, NPQ relaxation rate constant k. CT2: NPQ induction amplitude, NPQ induction rate constant k, NPQ relaxation rate constant k. CT3: Max NPQ, NPQ induction rate constant k, NPQ relaxation rate constant k. CT4: PI, $\Phi PSII$ recovery amplitude, $\Phi PSII$ recovery rate constant k.

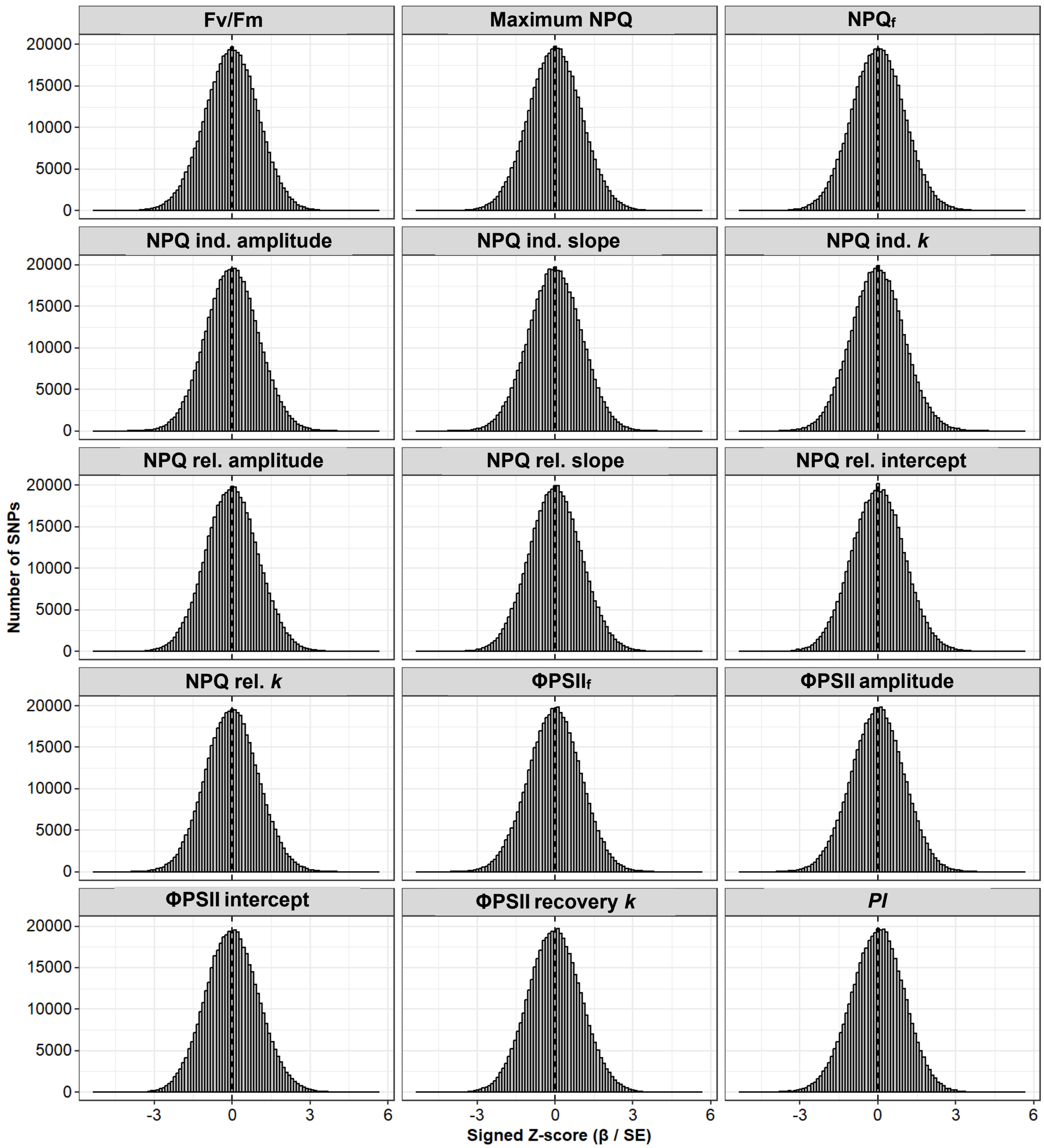

Figure S23: Signed Z-score histograms of 2017 univariate genome-wide association analyses, faceted by trait.

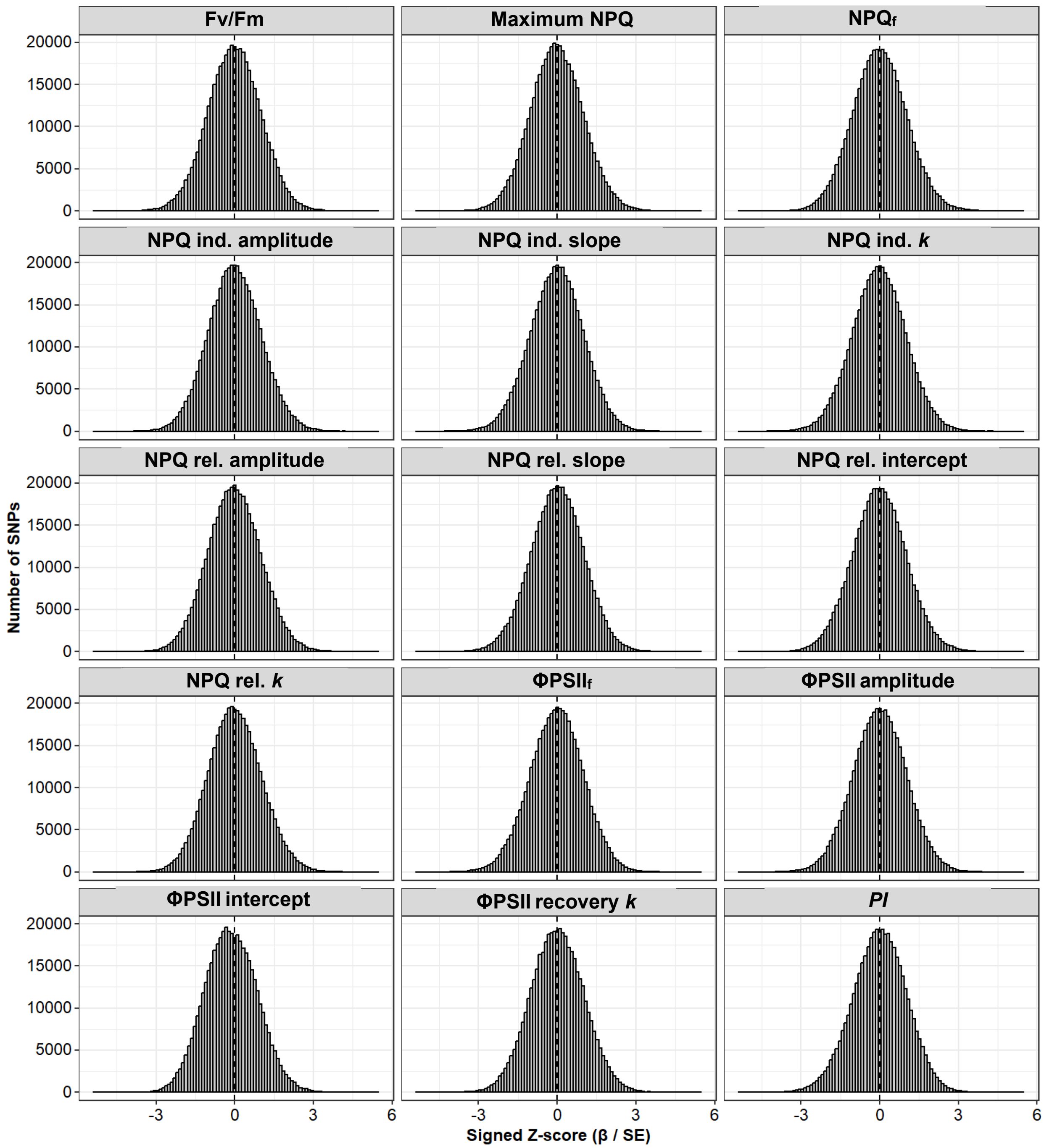

Figure S24: Signed Z-score histograms of 2019 univariate genome-wide association analyses, faceted by trait.

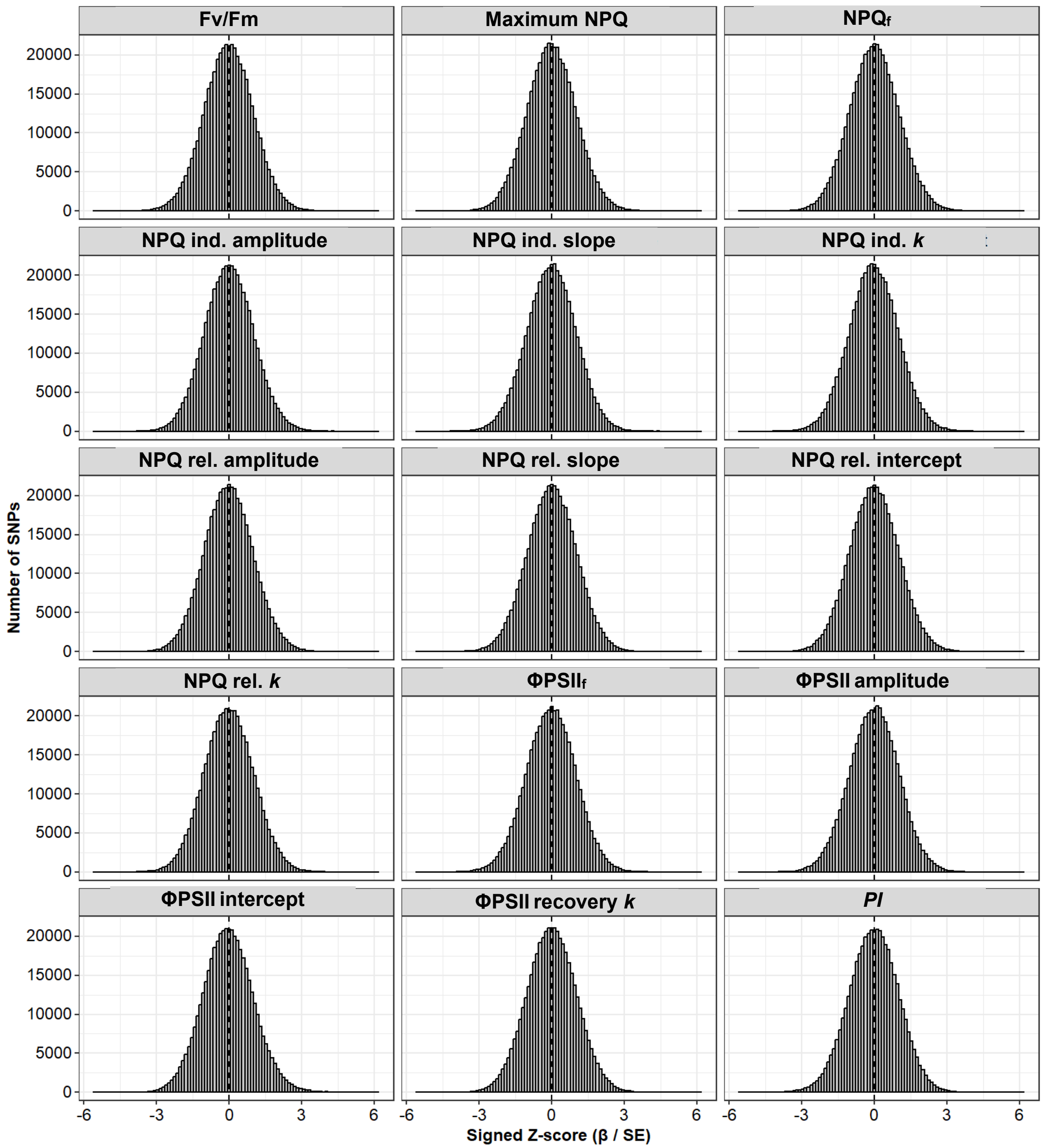

Figure S25: Signed Z-score histograms of joint univariate genome-wide association analyses, faceted by trait.

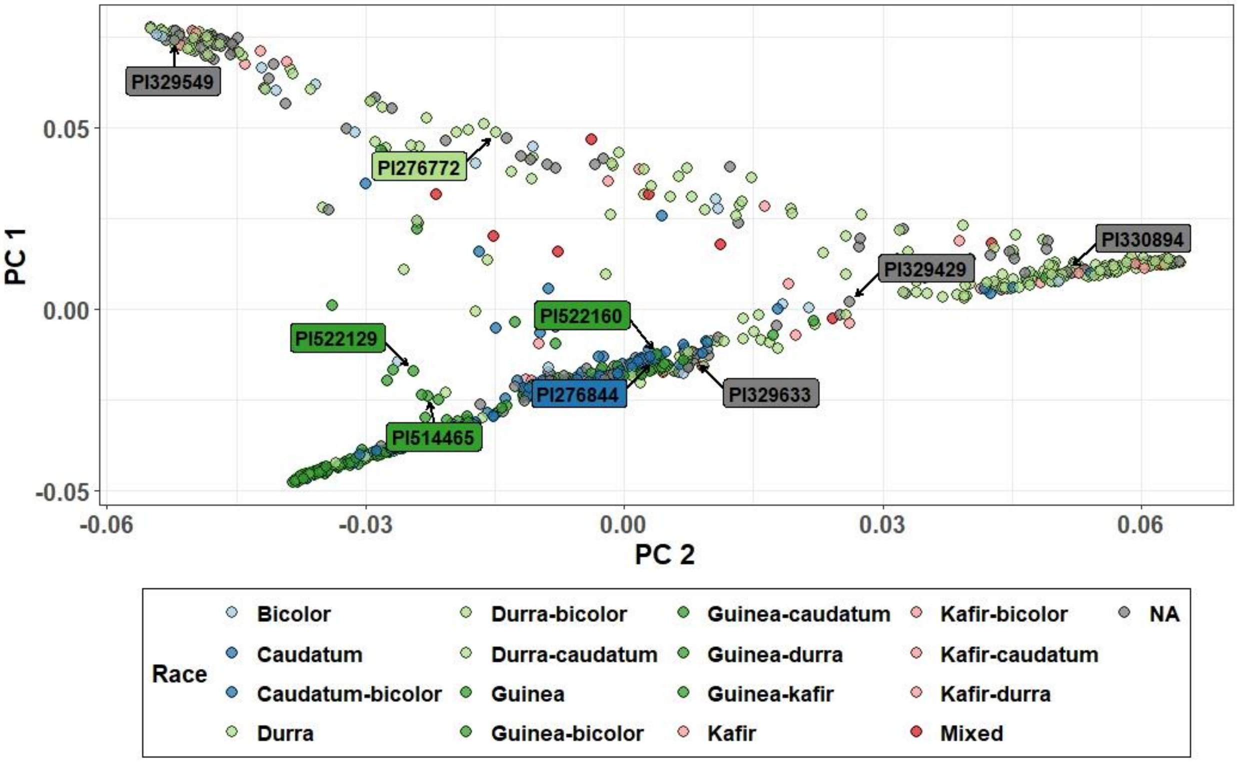

Figure S26: Biplot of first two components of principal component analysis of single nucleotide polymorphism allele values for 829 sorghum accessions. Colour denotes origin race. Trait space position of nine "top accessions" by Multi-trait Score are noted in boxes.
